# Use of Electrospinning for Sustainable Production of Nanofibers: A Comparative Assessment of Smart Textiles-Related Applications

**DOI:** 10.3390/polym16040514

**Published:** 2024-02-14

**Authors:** Marina Stramarkou, Ioannis Tzegiannakis, Erifyli Christoforidi, Magdalini Krokida

**Affiliations:** Laboratory of Process Analysis and Design, School of Chemical Engineering, National Technical University of Athens, 9 Iroon Polytechneiou St. Zografou Campus, 15780 Athens, Greece; jtzegiannakis@chemeng.ntua.gr (I.T.); echristoforidi@chemeng.ntua.gr (E.C.); mkrok@chemeng.ntua.gr (M.K.)

**Keywords:** electrospinning, protective clothing, green matrices, biopolymers, shape memory, antimicrobial protection, waterproof, breathability

## Abstract

Textile production is a major component of the global industry, with sales of over USD 450 billion and estimations of an 84% increase in their demand in the next 20 years. In recent decades, protective and smart textiles have played important roles in the social economy and attracted widespread popularity thanks to their wide spectrum of applications with properties, such as antimicrobial, water-repellent, UV, chemical, and thermal protection. Towards the sustainable manufacturing of smart textiles, biodegradable, recycled, and bio-based plastics are used as alternative raw materials for fabric and yarn production using a wide variety of techniques. While conventional techniques present several drawbacks, nanofibers produced through electrospinning have superior structural properties. Electrospinning is an innovative method for fiber production based on the use of electrostatic force to create charged threads of polymer solutions. Electrospinning shows great potential since it provides control of the size, porosity, and mechanical resistance of the fibers. This review summarizes the advances in the rapidly evolving field of the production of nanofibers for application in smart and protective textiles using electrospinning and environmentally friendly polymers as raw materials, and provides research directions for optimized smart fibers in the future.

## 1. Introduction

Textiles are one of the fundamental elements of human life, but they have remained in their conventional “passive” state for many decades [1]. Nonetheless, in the face of escalating global competition, the textile industry may find it challenging to maintain its current status, prompting the need for more innovative technologies to fabricate high value-added products [2]. Smart and protective textiles represent a transformative intersection of traditional textiles and technology.

Starting with smart textiles, these innovative fabrics have the ability to respond to different physical stimuli, such as mechanical, electrical, and thermal, and interact with their environment [1]. This is achieved by incorporating various technologies, such as sensors, actuators, and other electronic components, thus enhancing their functionality and opening up new possibilities for diverse industries, such as fashion, sports, healthcare, and the military [1,2,3].

**Figure 1:**
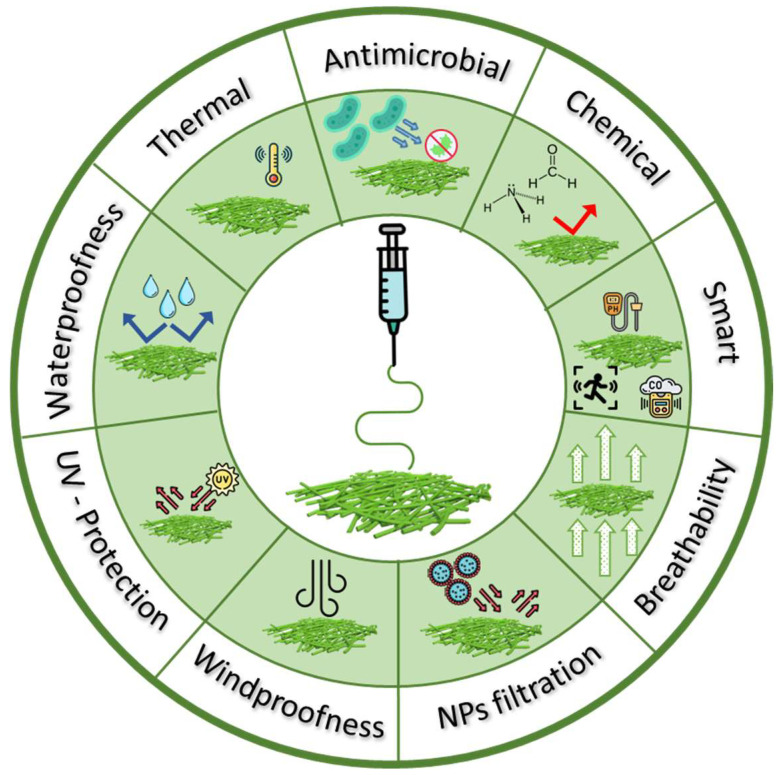
Types of properties of electrospun textiles prepared by bio-based and biodegradable polymers.

In parallel, protective textiles represent specialized fabrics designed to protect individuals from various external hazards or risks. These textiles are engineered to offer a range of protective functions, varying from resistance to heat, flames, chemicals, microorganisms, UV light, and hazardous particles to protection against wind and water [4,5], as shown in Figure 1. The development of protective textiles continues to evolve, incorporating technological advancements to enhance safety and comfort across various professional and recreational contexts.

In the traditional textile industry, conventional fabrics are made of fibers with high diameters (at least several microns or more) from passive materials, such as nylon and cotton [1]. With the development of science, nanotechnology has attracted a great deal of attention, being able to fabricate fibers that are sub-micron in diameter [2]. Among the numerous methods in the nanotechnology field, electrospinning plays a pivotal role in smart textiles. Electrospinning is a versatile and precise method for fabricating microscale to nanoscale materials from polymeric solutions based on electrohydrodynamics [6]. This technique not only enables the creation of controllable structures, providing a high surface area and porosity, but also incorporates functional elements into the fabric matrix [3,7,8,9].

Considering the fact that the textile industry is the second-largest polluting industry, globally contributing to 3% of total greenhouse gas emissions, and recognizing that the dominance of synthetic fibers over biopolymers is unsustainable due to the depletion of oil sources and growing environmental concerns, bio-based polymers are now starting to be employed in various textile processes [3,10]. The combination of electrospinning with bio-based polymers has the potential to completely transform the textile sector by lowering its environmental impact and bringing in a new era of sustainable, high-performance fabrics.

This review summarizes the advances in the rapidly evolving field of the development of nanofibers for smart and protective textile applications using electrospinning and environmentally friendly polymers as matrixes and/or additional agents. In addition, in the first Section, the review covers the fundamentals of electrospinning. More specifically, the working principles of this technology are elucidated and an overview of the electrospinning methods and process parameters that influence and tailor the properties of the electrospun fiber assemblies that make them suitable for textiles is provided. In addition, a general trend of the use of biopolymers is shown, highlighting the perspective on the potential future of the field.

### 1.1. Basics of Electrospinning

Electrospinning has been recognised as an efficient and simple technique for the fabrication of polymeric fibers that are sub-micron in diameter in terms of versatility, flexibility, and ease [2,11]. Thanks to the unique properties of electrospun fibers, which include their large surface area, high porosity, flexibility in surface functionalities, and good interfacial adhesion, they are showing promise for exploitation in textiles and clothing, filtration, and tissue and biomedical engineering [11,12].

**Figure 2:**
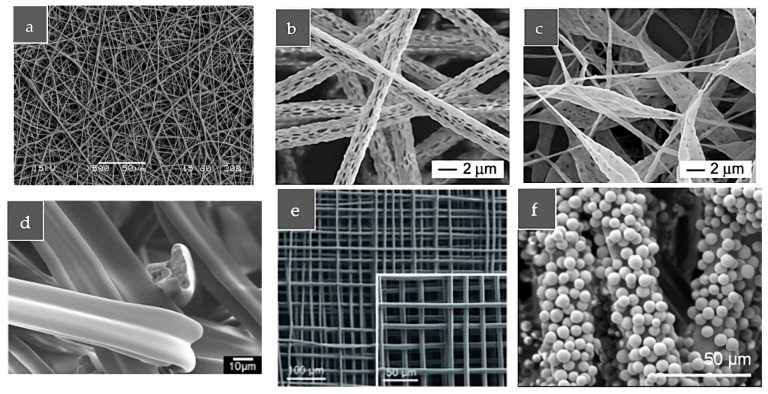
SEM photos of (**a**) randomly oriented and uniform electrospun PLA fibers, (**b**) highly porous, uniform electrospun PLA fibers [14], (**c**) beaded porous electrospun PLA fibers [14] (reproduced with permission from Xie et al. copyright 2008, John Wiley and Sons), (**d**) y-shaped CA electrospun fibers [15] (reproduced with permission from Chattopadhyay et al. copyright 2016, Springer Nature), (**e**) melt electrospun PCL fibers accurately deposited using an automated stage as the collector [13] (reproduced with permission from Brown et al. copyright 2011, John Wiley and Sons), and (**f**) electrospun PCL fibers coated with electrosprayed PLGA particles [16] (reproduced with permission from Bock et al. copyright 2014, John Wiley and Sons).

The produced fibers may have different diameters, ranging from several nanometers to several micrometers, different orientations, varying from random (Figure 2a) to accurate (Figure 2e) [13], and different shapes, varying from highly porous (Figure 2b) [14] to beaded (Figure 2c,f) [13,14] and y-shaped (Figure 2d) [15].

Typically, an electrospinning setup consists of (i) an injection pump, which permits the controlled volume feed of a polymer solution; (ii) a high-voltage power supply; (iii) a needle connected to a syringe and positive voltage; and (iv) a surface collector connected to negative voltage (Figure 2a). Briefly, during electrospinning, the polymer solution is charged by applying a high voltage. As the solution drop approaches the needle’s tip, the charges that accumulate on its surface cause it to lose its hemispherical shape and convert into a Taylor’s cone. When the electric field reaches a critical value, at which the repulsive electric force surpasses the surface tension of the polymer solution droplet, the polymer solution is ejected from the tip of the Taylor’s cone. On its route to the collector, the solvent evaporates and the jet solidifies, forming ultrafine fibers that are deposited on the collector (Figure 2a) [17,18,19].

### 1.2. Types of Electrospinning

There are many different types of electrospinning based on: (i) the type of the polymeric matrix (solution or emulsion), (ii) the type of nozzles and axial units (coaxial, monoaxial, or multiaxial), and (iii) the number of nozzles (single or multi-nozzles). These types determine not only the efficiency of the process and the encapsulation, but also the shape and the quality of the produced nanofibers [20].

**Figure 3:**
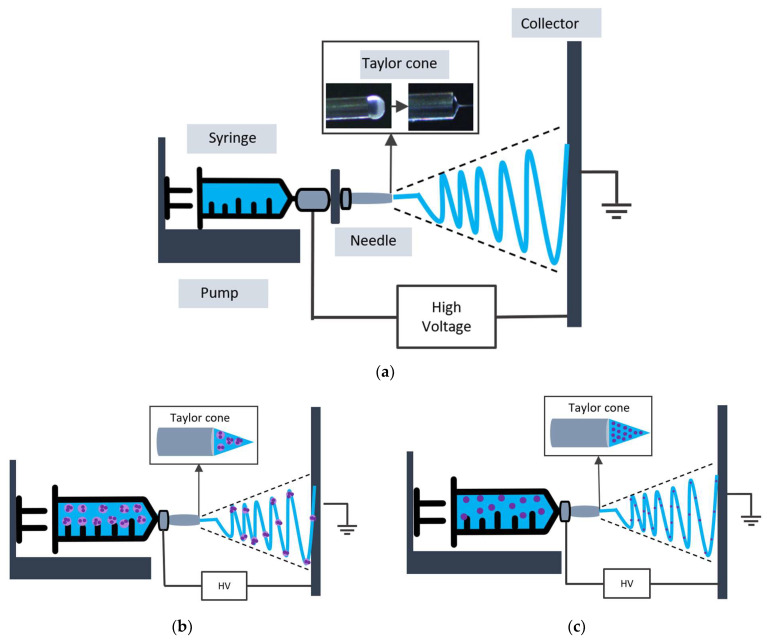

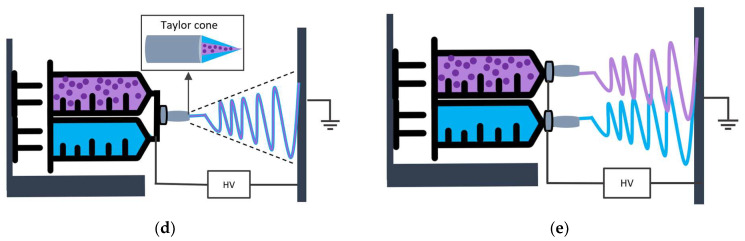
Set up of (**a**) conventional, (**b**) emulsion, (**c**) uniaxial, (**d**) coaxial, and (**e**) multi-nozzle electrospinning techniques. The solution of blue color represents the carrier material and purple color represents the compounds of interest to be encapsulated through electrospinning.

While in uniaxial electrospinning, the polymer, including the compound of interest (purple color in Figure 3c) and the carrier material (blue color), is mixed previously and ejected using a single needle, coaxial electrospinning is based on the simultaneous co-spinning of the two polymeric liquids from two different needles (Figure 3d) [21]. Therefore, the spinneret forms a composite polymeric droplet: the inner liquid (core) is pumped through the internal needle, and the carrier material (shell) through the external needle [22]. In addition, emulsion electrospinning is based on a core–shell structure formation by electrospinning a stable emulsion of two or more phases (Figure 3b). Finally, the multi-nozzle electrospinning assembly has the advantage of better productivity (Figure 3e) [19,20]. The most used electrospinning configurations, namely the conventional, the emulsion, the uniaxial, the coaxial, and the multi-nozzle electrospinning set ups, are depicted in Figure 3.

### 1.3. Parameters of Electrospinning

To fully explore the potential of electrospinning and develop nanofibers with useful applications in textiles, it is essential to acquire the desired morphology and specific diameter, as they critically influence the properties of the final product [23]. This can be achieved through thorough understanding of the electrospinning parameters and their effect on the properties of the fibers. These parameters can be classified into three principle categories: (i) the *solution parameters* that mainly include the polymer molecular weight, the polymer concentration in the solution, the solvent selection, the solution and interfacial viscosity, the surface tension, and the conductivity, (ii) the *processing parameters* that include the flow rate, the applied voltage, and the distance from needle tip to collector, etc., and (iii) the *ambient parameters*, such as the temperature and relative humidity [12,24,25]. The abovementioned parameters are grouped and their correlations with the morphology of electrospun fibers are summarized in Table 1.

In general, an increase in the value of the solution parameters leads to the formation of more uniform fibers and reduces the tendency to form beads and droplets during electrospinning, except for the surface tension, where the correlation between fiber morphology and surface tension is not clear yet. Therefore, the rheological characterization of the prepared electrospinning solutions can be applied as a predictive tool for the formation of fibers [3,24,26,27,28].

Regarding the processing parameters, they significantly depend on the polymer system. However, some general conclusions are that a moderate increase in the flow rate leads to the fabrication of thicker nanofibers, since the quantity of the polymeric matrix is higher, whereas an increase in the applied voltage and the tip-to-collector distance decreases the fibers’ diameter due to higher repulsive electrostatic forces and greater droplet “flight”, providing more time for Coulomb explosions and the vaporization of the liquid, respectively [24,29]. Finally, with regard to the ambient parameters, while an elevation in temperature clearly results in thinner fibers because of the solvent evaporation, the effect of the relative humidity displays a complex interplay and is influenced by the polymer system [24].

### 1.4. Types of Polymeric Matrices

Several synthetic and natural polymers, including single and blended polymers, have been used for the fabrication of the electrospun fibers that are applied in the manufacturing of protective and smart clothing thanks to their useful properties [17]. However, given the continuous efforts towards decreasing dependency on petroleum-based plastics and developing eco-friendly products in the last decades, bio-based polymers are preferred and explored by many researchers [30]. Bio-based polymers can be classified into non-biodegradable bio-based polymers, such as polyethylene terephthalate (PET), polyethylene (PE), polyamide (PA), and polypropylene (PP), etc., and biodegradable polymers, including PBAT, PBS, PLA, PHB, cellulose acetate, and chitosan, etc. [31].

**Figure 4:**
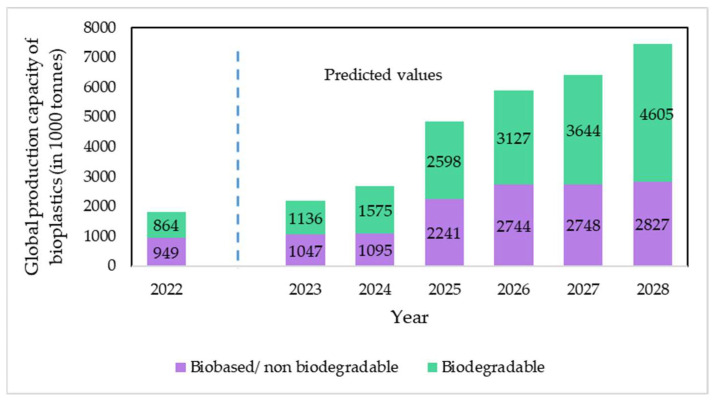
Evolution of global production capacities of bio-based polymers from 2022 to 2028 (in 1000 tons) [31].

In fact, according to the latest available data on European Bioplastics, due to the rising demand for more sophisticated and eco-friendly products, the global production capacity of bioplastics is predicted to increase significantly within the next 5 years, from 2.18 million tons in 2023 to 7.43 million tons in 2028, as seen in Figure 4 [31]. Bio-based polymers are used for an increasing variety of applications; however, fibers (woven and non-woven) and packaging are the two largest market segments for bio-based plastics with 26 and 24 percent of the total bioplastics market in 2022, respectively [31,32]. Nevertheless, the fabrication of bio-polymeric fibers for yarns can be complicated, since many bio-polymers require non-thermal processes in order not to denaturalize, such as electrospinning [30].

All the aforementioned data highlight the importance of a thorough review on the combination of electrospinning—as a promising and scalable method—and bio-based polymers. This synergy aims to produce the next generation of textiles that go beyond the conventional terms of protection, providing enhanced protective and smart features, but also prioritize environmental friendliness. In the scope of this article, a systematic review is performed on the studies and developments on protective and smart textile fibers made by electrospinning in conjunction with polymers from natural sources or with biodegradable characteristics.

## 2. Electrospun Fibers Used for Smart and Protective Clothing

The intersection of electrospinning technology and bio-based polymers has given rise to a fascinating array of smart and protective textiles that are not only technologically advanced, but also contribute to environmental conservation. In this Section, we will cover, in detail, the distinct categories of protective and smart bio-inspired textiles that emerge from the synergy between electrospinning and bio-based polymers. From materials designed to provide an extra layer of protection to responsive fabrics capable of adapting to environmental stimuli, the following discussion will cover a wide range of applications that cut across sectors like healthcare, sports, and industrial safety.

### 2.1. Antimicrobial Protection

In the past few years, an attempt has been made to establish green matrices for the production of electrospun fibers with plenty of properties and to replace the old conventional polymers, such as Nylon 6, PVDF, PEG, and PAN, etc., with the same capabilities. The antimicrobial effect of bio-based polymeric electrospun mats was measured most of the time against the most well-known Gram-positive and Gram-negative bacteria, *Staphylococcus aureus* (*S. aureus*) and *Escherichia coli* (*E. coli*), respectively, which can cause surgical site infections and infections after burn injuries, etc.

#### 2.1.1. PU-Based

One of the most used bio-based polymeric matrices for the production of micro and/or nanofibers with antimicrobial activity is PU. Due to the fact that pure PU does not exhibit antimicrobial efficiency, the encapsulation of various antimicrobial components in this matrix through the electrospinning process is required [33,34,35,36].

Ag NPs are one of the most widespread antimicrobial agents thanks to their interaction not only with the cell wall enzymes, but also with the DNA of bacteria, which leads to the end of DNA replication [34,37]. Starting with Sheikh et al. (2009), they tested the effectiveness of Ag NPs on *E. coli* and *Salmonella typhimurium* and discovered a great antibacterial performance, which was also confirmed by Pant et al. (2019) and Lakshman et al. (2010), who applied their PU/Ag NPs electrospun films on *E. coli*, *S. aureus*, and *Klebsiella* bacteria, respectively [33,35,37].

In addition, the combined use of Ag NPs with other antimicrobial agents in PU matrices was examined in order to investigate the possibility of antimicrobial activity enhancement. Ag NPs, together with GO, were encapsulated in PU, and it was proven that the combination of these agents showed the best antimicrobial effectiveness, killing 79% of *E. coli* bacteria. This was due to the fact that, in general, GO generates reactive oxygen species and leads to bacterial cell death [34]. Furthermore, Mamtha et al. (2023) used Ag NPs combined with Magnesium oxide (MgO) and achieved a remarkable diameter of inhibition zone equal to 21 mm and 30 mm for *E. coli* and *S. aureus*, respectively [38].

Τitanium dioxide (TiO_2_) NPs are another antimicrobial agent that were encapsulated in a PU matrix by Ryu et al. (2013) and killed 99% of *S. aureus* bacteria [39]. Moreover, Viscusi et al. (2023) encapsulated copper (Cu) NPs, zinc (Zn) NPs, and a combination of these in PU by electrospinning. The best results were reached in the case of PU/Cu, with 98% growth inhibition of *E. coli* and *S. aureus* [40].

#### 2.1.2. CS-Based

On the other hand, Mohraz et al. (2019) did not encapsulate any antimicrobial agent, but electrospun a blended matrix of PU and chitosan (CS), a natural polymer known for its excellent antimicrobial properties. It was observed that the polymeric blends with the larger inhibition zones of *E. coli* were those with the highest quantity of CS. Therefore, among the various PU/CS ratios of 95/5, 90/10, and 85/15, the latter was the best performing [36]. CS was also studied in the work of Desai et al. (2009) along with PEO, forming a blend in a ratio of 90:10 (*w*/*w*) to reach a satisfactory *E. coli* reduction of >2 log in 6 h. While the pure PEO mat had 0 log reduction, the addition of CS provided antibacterial action thanks to the NH_3_^+^ that bonds with the negatively charged elements of the cell wall and obstructs cell growth, while leading to its death [41]. Lin et al. (2020) used a pure CS: Hydroxypropyl cellulose (H): PEO (P) blend, coded as CSHP, as well as a CSHP blend with graphene (G) as an antimicrobial agent to compare their antibacterial capacity. The outcomes demonstrated that pure CSHP had an inferior performance in comparison with CSHPG, despite the great antimicrobial capability of CS and H in the pure matrix, which minimized the bacteria’s ability to attach on surfaces because of their hydrophilicity or hydrophobicity [42].

CS has also been frequently combined with PVA. PVA represents a commonly employed biodegradable polymeric matrix for electrospinning processes and, despite the fact that it has no antibacterial effectiveness as a pure matrix, it appears to have outstanding results in blends with other polymers and additives [17,43,44,45]. Vongsetskul et al. (2015) examined the antimicrobial effectiveness of a trimethylated CS (TMCS)-loaded PVA blend and pure PVA, with the latter having no antimicrobial effect. The TMCS:PVAI blend performed well against *E. coli*, *Pseudomonas aeruginosa*, *Acinetobacter baumannii*, and *Candida albicans* due to the bonding of TMCS with the lipoteichoic acids of the Gram-positive bacteria cell walls and the lipopolysaccharide of the Gram-negative bacteria outer membrane [43].

In addition, Park (2010) and Yang et al. (2018) tested the properties of PVA:CS mats with encapsulated Ag and GO, respectively, and measured satisfactory activities against *E. coli* and *S. aureus* for various polymer: antimicrobial agent ratios [17,45].

#### 2.1.3. PVA-Based

PVA has been also studied as a matrix during electrospinning along with other agents, except for CS. Purwar et al. (2016) used PVA with sericin and clay and noticed that, by increasing the quantity of clay, the antibacterial capacity was improved, with the 0.75% clay concentration leading to the death of 98.3% of *E. coli* and 97% of *S. aureus* [46]. In addition, Lee and Lee (2012) and Khan et al. (2019) examined the properties of PVA using various concentrations of TiO_2_ and ZnO NPs as antimicrobial additives, respectively. The first one had to activate the TiO_2_ NPs by UV irradiation for 3 h in order to test the PVA:TiO_2_ antimicrobial activity, which was equal to 99.3% against *S. aureus* (with 2 wt% TiO_2_) and 85.3% against *Klebsiella pneumoniae* (with 3 wt% TiO_2_). The poorer outcomes in the case of *Klebsiella pneumoniae*, even with a higher TiO_2_ concentration, were due to the extra complexity of the Gram-negative bacteria cell wall structure (two lipid bilayers) [47]. Furthermore, Khan et al. (2019) tested the PVA: ZnO antimicrobial effectiveness, which was improved by increasing the quantity of ZnO additive [44]. Khanzada et al. (2020) and Zhang et al. (2020) encapsulated Aloe Vera (AV) and Quaternary ammonium salt (IQAS) in PVA matrices, respectively. AV was selected as the antibacterial agent against *S. aureus* and *E. coli*, reaching good inhibition zones thanks to its anthraquinones and pyrocatechol [48]. Finally, in the work of Zhang et al. (2020), where IQAS was encapsulated in PVA, the findings revealed that, by increasing the quantity of IQAS, the antibacterial effect of the matrix was upgraded, leading to 99% reductions for both *S. aureus* and *E. coli* [48].

#### 2.1.4. PCL-Based

PCL represents another biodegradable polymer appropriate for use as a matrix in electrospinning for the encapsulation of additives, which display great antimicrobial properties. Paneva et al. (2011) examined the cases of PCL:Ascorbyl Palmitate (AP) and PCL:AP:Ag NPS blends, and was led to the conclusion that both electrospun blends exhibited similar inhibitions of *S. aureus* bacteria [49]. It was also proven that neat PCL showed no antimicrobial activity [49,50]. Augustine et al. (2014) and Permyakova et al. (2022) studied the inhibition zones of PCL: ZnO NPs mats against *S. aureus* and *E. coli*, with the first one concluding that, due to the damage on the bacteria cells that occurred by hydrogen peroxide, the inhibition zones had diameters of 10.22 ± 1.3 mm and 9.81 ± 0.8 mm, respectively [50]. Ghosal et al. (2021) took advantage of the antimicrobial properties of hydrophobic carbon quantum dots (hCQDs), which ruined the bacterial cell wall thanks to reactive oxygen species (ROS), leading to significant antimicrobial effects against *S. aureus*, *Listeria monocytogenes*, *E. coli*, and *Klebsiella pneumonia* [51]. Finally, Costa et al. (2021) fabricated PCL: MgO NPs mats via electrospinning and noticed a satisfactory performance against *S. aureus* and *E. coli* due to the generation of reactive oxygen species, which destroyed the bacterial cell [52]. For the same bacteria, Zhou et al. (2021) concluded that the electrospun PCL:CS oligosaccharides (COS):Quercetin (Qe) showed a great antimicrobial capacity [53].

#### 2.1.5. CA-Based

Furthermore, the antimicrobial properties of cellulose acetate (CA), the most abundant renewable polymer on Earth and naturally found as a fibrous structure, were examined against *S. aureus* and *E. coli* [54,55,56]. Jatoi et al. (2019) produced CA:ZnO:Ag NP fibers with great inhibition thanks to the generation of reactive oxygen species [54], whereas Beikzadeh et al. (2020) encapsulated lemon myrtle essential oil (LMEO) in a CA matrix and achieved a 100% elimination of these bacteria [55]. Li and Yang (2020) encapsulated rutin in a CA:PEO blend matrix, resulting to a 97.2% inhibition of *S. aureus* and 98.5% of *E. coli* [56].

#### 2.1.6. Other Polymers

Beyond the most commonly used matrices, there are plenty of others that were utilized to enhance the antibacterial action of the fibers, including Poly(Butylene Succinate) (PBS), Poly(3-ydroxybutyrate) (PHB), PBAT, PLA, and gelatin. Tian et al. (2013) produced both pure electrospun Poly(Butylene Succinate) (PBS) fiber mats and PBS with encapsulated Ag NP fiber mats. Pure PBS demonstrated zero inhibition growth, while encapsulated Ag NPs showed enhanced long-term antimicrobial capabilities against *S. aureus* and *E. coli*, with 99% inhibition for both bacteria [57]. On the other hand, PHB electrospun fibers were examined by Rodríguez-Tobías et al. (2016) against the same bacteria, with the findings indicating that neat PHB fibers increased the bacteria population, while electrospun PHB with ZnO NP additive fibers attained great bacteria inhibition [58]. Duygulu et al. (2020) evaluated the antimicrobial efficacy of PLA with ceftriaxone disodium electrospun fibers against plenty of microorganisms, such as *Bacillus cereus*, *Listeria monocytogenes*, *E. coli*, and *Salmonella Typhimuriume* and noticed enhanced inhibition zones for the drug-blended PLA nanofibers [59]. Great antimicrobial capabilities against *E. coli* were also achieved in the cases of Silk Fibroin (SF)/gelatin (G) fibers with encapsulated GO and Ag NPs [60], as well as electrospun PHBV and PBAT blended mats with TiO_2_ NPs [61].

All the above-mentioned electrospun films with antimicrobial protection are summarized in Table 2, where the materials used for their production, the optimum processing conditions, their structural characteristics, and their potential applications are also indicated. 

### 2.2. Micro-Nanoparticle Filtration

The intended use of electrospun fibers in textile applications leads to the examination of their air filtration capability, so that they can effectively filter the micro/NPs of the atmosphere, such as PM 2.5 (particle matter of 2.5 μm or less), PM 10 (particle matter of 10 μm or less), and smoke components, which may create hygiene issues in inhalers. PM 2.5 are fine particles that can enter respiratory tracts and proceed deeper into the lungs, where their deposition could cause tissue damage and lung inflammation [64]. On the other hand, PM 10 are bigger particles, like dust from roads and farms, that cannot reach the inside of the lungs; however, they induce headaches and great discomfort in the eyes, noses, and throats of people near them, while, at the same time, a significant amount of PM 10 are deposited into their larger airways [65]. To prevent these health problems, it is essential to fabricate textiles that show a sufficient filtration efficiency and provide protection for the wearer, while, at the same time, have a great biodegradation and renewability ratio, following the eco-friendly world consciousness, so that they can be extensively used in facemasks and protective suits. Electrospinning is employed as a method to produce these biodegradable textiles due to its ability to create nanofiber mats with a low porosity, able to trap harmful NPs. The air filtration effectiveness of nanofiber mats depends on the different matrix blends and optimum condition factors that are used by impacting characteristics, such as the porosity of the fiber, its diameter, and the filter thickness [52].

#### 2.2.1. CS: PEO-Based

One of the first matrix blends used for the fabrication of electrospun nanofiber mats with high filtration quality was High-Molecular-Weight (HMW) CS: PEO, which was studied in the work of Desai et al. (2009), where it was concluded that, by increasing the CS content, the electrospinning fibers’ aerosol filtration efficiency was improved, due to a higher metal binding rate. The impact of the nanofibers’ diameter on the filtration efficiency was also studied, and it was proven that, by increasing it, the filtration efficacy was decreased due to the enlargement of the fiber mat pore size [41].

#### 2.2.2. PVC: PU-Based

Some years later, Wang et al. (2013) indicated the importance of the optimum ratios of the matrices in the blends used for electrospinning. It was revealed that, by increasing the PU content in PVC: PU blends, the filtration of the fiber mats was enhanced due to an increase in the average fiber diameter, which may have occurred from the rise in viscosity and decline in the solution conductivity. At the same time, a great increase in the PU concentration may lead to enhanced adherence between the nearby fibers, which is not desirable for fiber mat structures. This fact highlights the importance of finding the optimum process conditions during electrospinning, such as the blended matrix ratio, voltage, feed rate, and tip-to-collector distance, in order to not only increase the fiber diameter and therefore the filtration efficiency, but also maintain a proper fiber structure. By finding these optimum process conditions, Wang et al. (2013) achieved a high air filtration percentage equal to 99.5% towards NaCl APs with a diameter of 300–500 nm [66].

#### 2.2.3. CA-Based

CA represents another matrix used neat or blended with other materials for the creation of electrospun fibers with air filtration capability. Chattopadhyay et al. (2016) developed CA nanofiber filters and studied the parameters, such as fiber diameter and filter thickness, that affect the filter performance and clogging against NaCl (solid AP) and diethyl hexyl sebacate (DEHS) (liquid AP). The ideal filter thickness was selected based on a tradeoff between the mechanical integrity of the mat and the quality factor for aerosol filtration, and the overall study proved that the electrospun filters demonstrated slightly better-quality factors compared to commercial glass fiber filters, but at a much lower thickness [15]. Motivated by the eco-friendliness of CA, De Almeida et al. (2020) fabricated electrospun fibers of CA: Cetylpyridinium Bromide (CPB) and observed that the addition of CPB did not affect the filtration percentage of the fabricated mats, but enhanced other capabilities, such as the antibacterial activity [67].

#### 2.2.4. PLA-Based

PLA is a matrix with intense interest due to its superb biodegradable rate and good mechanical strength. PLA’s renewability, combined with its outstanding spinnability for the development of electrospun nanofibers, is a promising sign for replacing other conventional matrices, such as polypropylene, that are used for textiles with air filtration properties [68]. Z. Wang et al. (2015) used PLA in different concentrations to develop fiber mats through electrospinning and noticed that their filtration efficiency was affected by the PLA concentration because of the variations in the fiber diameter. Specifically, small concentrations of PLA (5 wt%) led to thin fiber diameters (273.6 nm) and an outstanding filtration efficiency towards NaCl AP with a 260 nm diameter, which was appropriate for face mask filter applications [69]. Lo et al. (2022) also used neat PLA matrices to produce textiles for healthcare apparel and protective equipment. Even though the mean diameter of the developed nanofibers was 2.4 times larger than that of Z. Wang et al. (2015), they showed excellent filtration effectiveness towards APs of 0.1 μm when they were used as a double layer in face masks [70]. Furthermore, Zhang et al. (2019) created piezoelectric PLLA and PDLA fibers by electrospinning, utilizing the technology of electrostatic charge-assisted air filtration and valorizing the fact that the electrostatic force generated from the PLLA nanofibers can benefit submicron particle absorption. Actually, the fibers showed a 99% filtration efficiency for PM 2.5 particles and a 15% improvement in quality factor compared to a 3M commercial respirator filter, proving their potential for personal protective equipment applications [71]. Finally, Buluş et al. (2020) encapsulated activated carbon (AC) in PLA mats through electrospinning and their combination led to outstanding bacterial and submicron particle filtration efficiency, since AC can separate not only volatile organic compounds (VOCs), but also odors (e.g., cigarette smell) and gaseous pollutants from the air by capturing the molecules in coal beds [72]. 

#### 2.2.5. Other Polymers

Other interesting works studying the air filtration efficiency of electrospun fiber mats from green or recycled materials, such as PVA, PCL, PU, gelatin, and recycled PET, are presented followingly. Purwar et al. (2016) pioneered by investigating the filtration effectiveness of an electrospun film made of sericin and PVA with the additive of clay, acting as antimicrobial and reinforcing filler. The results of the study showed that, as the clay concentration was increased to 0.75%, the produced mats filtered a greater concentration of PM of 1 µm size, equal to 0.73 mg/m^3^/s, enabling them to be appropriate for air filtration mask applications [46]. PCL also represents another green biodegradable matrix examined for facemask applications. Costa et al.’s (2021) research revealed that neat PCL electrospun membranes have a good filtration efficiency equal to 84.90%. However, when NPs were added into the PCL matrix, such as Ag, TiO_2_, and MgO, a variation in the filtration effect was witnessed. Among the three studied NPs, MgO decreased the pore size of the electrospun fibers compared to pure PCL, resulting in an enhanced air filtration equal to 99.40% [52]. Continuing with PU, fiber mats were produced from neat PU and PU with CH and showed a great filtering efficacy not only for particle sizes smaller than 0.6 μm and higher than 0.6 μm, but also for 10–1000 nm KCL NPs, respectively [36,73] Furthermore, Souzandeh et al. (2016) examined an environmentally friendly electrospun nanofiber filter from gelatin. Gelatin is a natural protein with plenty of functional groups in its molecular structure that interact with several pollutants and particle matter, leading to fibers with impressive particulate removal efficiencies of more than 99.3% and 99.6% for PM0.3 and PM2.5, respectively [74]. Finally, Strain et al. (2015) fabricated electrospun fibers made from recycled PET and observed that, by increasing the PET content, the fiber diameter was increased and the filtration efficiency deteriorated. Thus, the fibers with the lowest PET concentration (10 wt%) showed a remarkable absorbed smoke content (43.7 times the weight of the fibers) and an air filtration efficacy of 99.99% towards NaCl APs with 260 nm diameter [75].

All the works on the electrospun films that provide micro- nano particle filtration are summarized in Table 3. 

### 2.3. Air Permeability

Breathable and waterproof fabrics have aroused both scientific and industrial attention in the field of protective and sports clothing, since they can effectively shield the human body from adverse external conditions, such as wind, heat, and water, while allowing water vapor to transfer from the inside to the outside atmosphere [76,77]. Breathability, also known as vapor transmission or air permeability, is a fabric’s ability to permit the transmission of moisture vapor by diffusion. This property facilitates evaporative cooling, ensuring the comfort of human skin [77,78]. The value of this ability is inversely correlated with waterproofness and closely associated with the application of the fabric and the comfort that it is supposed to provide. Fabrics with lower air permeability are primarily employed in protective clothing, whereas breathable fabrics offer great comfort and find promising applications in various fields like sportswear [79,80]. Consequently, the demand for the development of advanced breathable fabrics besides traditional textiles has given prominence to electrospinning as a method for producing this type of fabric [77]. Electrospun webs consist of fibers with very small diameters, micro and/or nano-pores, and a high surface area, providing excellent vapor release properties while preserving water and wind resistance. Therefore, they can be applicable to both waterproof and breathable fabrics [76]. However, designing such materials that integrate environmental friendliness and a high functionality has been considered as a longstanding challenge [78].

#### 2.3.1. PU-Based

A bio-based polymeric matrix with a wide range of desirable properties that is extensively examined and capable of displaying high and low air permeability, depending on its use case, is PU. PU has been studied in many works as a pristine matrix or blended with other matrices.

Kang et al. (2007) produced multi-functional fabrics by the direct electrospinning of PU onto substrate fabrics and evaluated their breathable properties in comparison with PU-resin-coated fabrics according to the ASTM D737 testing method. The electrospun webs exhibited higher air permeability compared with the resin-coated fabrics thanks to their many open-structure pores, proving that electrospinning can be used for the production of breathable fabrics [76]. The structural characteristics, and more specifically, the numerous nano-sized pores, were again the reason for the high air permeability of the stretched PU films prepared by Chung et al. (2011), with the aim of manufacturing intelligent clothing material [81,82]. In this regard, Gorji et al. (2012) explored the optimum process duration for preparing breathable windproof protective clothing based on neat PU electrospun nanofiber mats and concluded that shorter electrospinning processes led to thinner and lighter webs, and thus to improved air permeability [82]. This was in agreement with the work of Amini et al. (2016), who noticed that less processed PU films were thinner, had lower adhesion between the nanofibers, and a better air permeability in contrast to thicker films that showed pore tortuosity phenomena [79]. Several processing parameters were also investigated in the study of Sadighzadeh et al. (2016) in order to improve the waterproof and breathable properties of fabrics developed by applying electrospun webs of PU, PAN, and PES onto a substrate fabric. The air permeability of the three before-mentioned webs were compared to gortex, a commercial protective multilayered fabric, and the best-performing web was the PU one. This confirms that green biodegradable films are not only superior to their synthetic electrospun counterparts, but also to conventional fabrics used in sportswear, since they are elastomer enough to resist against wind and folding [77].

Except for plain PU electrospun films, various studies have focused on the use of blended PU matrices and/or the encapsulation of additional agents into them through electrospinning, so as to improve the breathability characteristics.

Starting with the encapsulation of additives in a pristine PU matrix, Gu et al. (2018) developed fiber films of PU with incorporated hydrophobic silica gel (HSG), and after their chemical and thermal post-treatment in order to transform HSG to hydrophilic, the breathability was improved by 25%. This was due to a better adhesion structure among adjacent fibers that allowed the molecular chains to expand and rearrange [80]. Moving to blended matrixes, Ge et al. (2013) incorporated an innovative synthesized FPU into PU to enhance the porous structure, whereas Wang et al. (2012) additionally incorporated SiO_2_ NPs into an FPU matrix solution in order to further increase the surface area of the developed superamphiphobic nanofibrous membranes, and therefore, their air permeability [83]. The fact that the morphology of the developed electrospun webs significantly affects their final properties was also confirmed in the study of Wang et al. (2013) and Gorji and Maryan (2018), where fibers of PVC/PU and PAMPS/PU were developed, respectively. Both studies concluded that, by increasing the PU content in the matrix, the air permeability was decreased due to the reduction in the open pores and the strongest adhesion among adjacent fibers [66,84].

Another interesting observation is that, sometimes, the post-treatment, more specifically the lamination of electrospun films, may lead to a lower air permeability, because the molecules of the laminant can block pores and render the treated surface hydrophobic, as in the case of Jin et al. (2016), who laminated PU/silica NPs with a non-fluorinated water repellent chemical, n-dodecyltrimethoxysilane (DTMS), and noticed a small reduction in air permeability compared to the non-laminated sample [85].

#### 2.3.2. Other Polymers

PU is a widely used matrix for the development of breathable fabrics through electrospinning; however, other green matrices have been also studied recently. Starting with Costa et al. (2021), they prepared biodegradable facemasks by functionalizing electrospun PCL fibers with MgO NPs and incorporating them in the middle of two cotton layers. This incorporation led to a decreased air permeability compared to the plain cotton facemask, as the weight and the thickness were increased and the porosity was decreased [52]. In addition, bicomponent membranes were produced by adding AlCl_3_ into two enantiomers of PLA, namely PDLA and PLLA, through side-by-side electrospinning in the work of Gao et al. (2021). The addition of salt resulted in both a reduction in the fiber diameter and pore size and an increase in the specific surface area, contributing to the membranes’ superior air permeability equal to 10.90 cm^3^/s/cm^2^ [86]. Finally, Fadil et al. (2022) conducted a very interesting preliminary study where PVA electrospun nanofibers were inserted as a filter between the layers of a facemask and provided superior properties over melt-blown fibers, such as a small pore diameter and fiber size, interspaces, and an improved air permeability equal to 28.55 cm^3^/s/cm^2^. Therefore, the application of active electrospun filters as a potential core component in facemasks can provide better protection from airborne threats [87].

All the above-mentioned works are summarized in Table 4, where the processing conditions for the production of the electrospun structures, as well as their morphological and air permeability characteristics, are presented and compared.

### 2.4. Water Vapor Breathability

Water vapor breathability is a crucial quality that sustainable protective textiles must possess in order for clothing to provide thermal comfort. Additionally, it prevents body overheating caused by metabolic reactions, skin irritation, and skin illnesses. Fabrics known for their breathability, such as cotton and linen, permit air and moisture diffusion, exhibiting a high water vapor transmission rate (WVTR). This means that an effective amount of water vapor is transmitted through the fabric per unit time [89].

#### 2.4.1. PU-Based

Various eco-friendly multifunctional mats have been fabricated via electrospinning, demonstrating a good WVTR without compromising other essential textile properties (Table 5). PU matrices have been extensively studied and commonly used in different forms, like plain PU, FPU, waterborne PU (WPU), silicon-based PU (Si-PU), and PU combined with other matrices and/or combined with different additional agents. Beginning with plain PU, nanofibrous webs exhibited a range of WVTRs, from a moderate WVTR of 40.67 g/m^2^/24 h with a 252.1 μm web thickness [82] to a good WVTR of 1720 g/m^2^/24 h with a 0.010 mm web thickness [79] or even a superior WVTR of 9200 g/m^2^/24 h due to increased fabric porosity [80]. Water vapor permeability happens through the fabric’s pores when moisture is absorbed by the fabric and then evaporated off its surface. Consequently, a very high thickness may influence water vapor permeability, reducing the web’s WVTR [79].

In many studies, PU electrospun webs have been directly applied onto a substrate fabric and still efficiently permitted water vapor transmission. Kang et al. (2007) produced a PU electrospun web on a polyester/nylon blended fabric, which exhibited a high WVTR of 9020 g/m^2^/24 h by selecting a relatively large average fiber diameter [76], while Yoon and Lee (2011) fabricated a two-layered fabric system of a PU nanofiber web onto densely woven polyester with a good WVTR at a low fabric thickness [90]. Similarly, Maksoud et al. (2018) utilized polyester screen fabric with a thickness of 250 μm and fabricated a two-layered system with PU nanofibrous webs with a lower WVTR of 1090 g/m^2^/24 h [73].

Many researchers have investigated the combination of PU matrices with their fluorinated form. Ge et al. (2013) fabricated PU/FPU electrospun microfibrous membranes with a large average fiber diameter of 995 nm, which increased proportionally with the concentration of FPU in the matrix solution. These PU/FPU membranes exhibited an excellent WVTR of 9210 g/m^2^/24 h and were suitable for diverse applications, such as protective clothing [88]. Y. Li et al. (2016) also utilized a PU/FPU matrix solution and the same solvent (DMF), but with higher concentrations of both PU and FPU in the matrix solution (almost five times higher). The result was a hydrophobic fibrous and porous membrane, which exhibited an even more superior WVTR of 11,900 g/m^2^/24 h [91]. In another study, lithium chloride (LiCl) was encapsulated in a PU/FPU matrix to create an electrospun fibrous membrane with an excellent WVTR of 10,900 g/m^2^/24 h. The higher LiCl concentration and resulting increased porosity created more passageways, facilitating more efficient water vapor diffusion [92]. Similar findings were reported by Y. Li et al. (2015), who encapsulated carbon nanotubes (CNTs) in a PU/FPU matrix to produce macroporous membranes with a WVTR of 9200 g/m^2^/24 h. The water vapor transmission rate increased with a higher CNTs concentration, attributed to the higher membrane porosity and the growing number of passageways [93]. More recently, Yue et al. (2021) fabricated nanofibrous membranes via a portable electrospinning device using an ethanol-soluble PU/FPU matrix and thymol as an additional agent. The membranes, compatible with human skin, exhibited a WVTR of 3560 g/m^2^/24 h, making them ideal for wound dressing due to their concurrent antimicrobial activity [94].

Plain PU matrices have been combined with various additional agents to either enhance already existing properties or introduce new features to the resulting garments. Starting with Jin et al. (2016), they encapsulated hydrophobic SiO_2_ SNPs in a PU matrix and created superhydrophobic webs with a large average fiber diameter via a one-step sol–gel electrospinning process directly on a polyester fabric. The presence of SiO_2_ SNPs in the matrix solution was found to have minimal impact on the WVTR of the fabric, which reached approximately 6160 g/m^2^/24 h after the process [85]. Similarly, P. Li et al. (2020) fabricated PU-SiO_2_ SNPs hydrophobic nanofibrous webs by selecting higher concentrations for both PU and SiO_2_ NPs in the electrospinning solution. The resulting webs had uniform fibers and exhibited a slight increase in WVTR of 8070 g/m^2^/24 h [95]. In a more recent study, Wang et al. (2022) utilized PU emulsion treated with 4,4′-methylenebis-(phenyl isocyanate) (4,4′-MDI) and 3-aminopropyltriethoxysilane (APTES), combined with SiO_2_ particles, which served as the additional agent. They fabricated superhydrophobic membranes with an average fiber diameter of 245 nm via electrospinning combined with a hydrothermal-assisted sol–gel method, resulting in membranes exhibiting an excellent water vapor permeability with a WVTR of 10,120 g/m^2^/24 h [9]. Noteworthy breathable properties were also observed by Zhao et al. (2018), who created eco-friendly fibrous membranes by introducing a novel PU elastomer (C4FPU) and AgNO_3_ into the PU polymeric solution. The C4FPU elastomer, containing a double-terminal short perfluorobutyl (−C4F9) chain, imparted durable hydrophobicity to the membrane, while AgNO_3_ decreased the maximum pore size, leading to a WVTR of 13,400 g/m^2^/24 h [96].

WPU matrices represent another commonly used type of PU, known for their environmentally friendly and safer characteristics. WPU is often combined with other water-soluble or water-dispersible polymers for emulsion electrospinning. In one study by W. Zhou et al. (2021), 0.6 wt% of PEO was initially dissolved in WPU emulsion, followed by the addition of polycarbodiimide (PCD) and long-chain alkyl polymer (LAP) emulsions (PCE and LAE). The result was the production of nanofibrous membranes with a WVTR of 4885 g/m^2^/24 h. A crosslinks network between PCD and WPU resulted in a stable interconnected porous structure of the membranes, ensuring effective water vapor permeability and making the waterborne fluorinated-free fibrous membranes capable for use in medical hygiene, wearable electronics, and other applications [8]. One year later, W. Zhou et al. (2022) created nanofibrous membranes through emulsion electrospinning that exhibited an even higher WVTR of approximately 13,000 g/m^2^/24 h. By incorporating trimethylolpropane tris (2-methyl-1-aziridine propionate) (TTMA), PEO, and water-based fluoropolymer emulsions (WFE) into a WPU matrix at optimum concentrations, they obtained nanofibrous membranes with excellent breathability, attributed to their high porosity structure [97].

In recent years, silicon-based PU (SIPU) matrices have also gained prominence for the production of electrospun fibrous membranes as fluorine-free, environmentally friendly materials. P. Zhang et al. (2022) fabricated siliceous PU nanofibrous membranes via electrospinning and examined the impact of stearic acid (SA) presence in the electrospinning solution. Despite SA being recognized as an inexpensive phase-change material, extensively used in various temperature-regulating materials, an increase in its concentration was found to slightly reduce the membrane’s porosity and, consequently, its water vapor permeability. As a result, nanofibrous membranes without SA exhibited a higher WVTR of 8900 g/m^2^/24 h [98]. In another study, a silicon-based PU matrix was combined with polymethylmethacrylate (PMMA), and an electrospinning process followed by UV irradiation was conducted to introduce octadecanethiol (thiol) and 2, 4, 6, 8-tetramethyl-2, 4, 6, 8-tetra-vinylcyclotetrasiloxane (TMTVSi) into the polymeric solution. After UV irradiation, the WVTR of the membranes was reduced due to decreased porosity, yet still maintained an effective WVTR [99]. Ren et al. (2023) incorporated PMMA into the matrix and as an additional agent to the polymeric solution, which contained 0.1 wt% of lithium chloride. The addition of polyvinyl butyral (PVB) to the electrospinning solution resulted in a noticeable reduction in the porosity of the produced membranes, leading to a decreased water vapor permeability. Consequently, nanofiber membranes with the highest examined WVTR of 9370 g/m^2^/24 h did not contain any PVB and were a result of an 80 °C heat treatment [100].

PU matrices are frequently combined with other polymers to create membranes that merge the properties of both components. For example, PAMPS, a hydrophilic and ionic polymer that forms super absorbent nanofibers, has been combined with hydrophobic PU nanofibers to create membranes with properties well-suited for protective clothing. Gorji and Sadeghian Maryan (2018) reinforced PU/PAMPS electrospun nanofibers by incorporating GO, resulting in nanofibrous membranes with a WVTR of 860 g/m^2^/24 h [84], while Gorji et al. (2018) improved this rate to 1430 g/m^2^/24 h, using a more concentrated solution of PAMPS [101]. The inclusion of GO is important, as it enhances membrane strength and stability and improves water vapor permeability, due to the formation of many hydrophilic groups that accelerate water vapor molecules [84]. Another example involves the combination of PU and PCL diol in the electrospinning solution to create PCL-based shape memory PU webs. These webs exhibited a WVTR of approximately 5900 g/m^2^/24 h, rendering them ideal for intelligent clothing materials [81]. In another study, Xu et al. (2017) integrated eco-friendly PU with PAN, a synthetic polymer, to produce nanofiber membranes with an excellent water vapor permeability of 12,900 g/m^2^/24 h. TiO_2_ NPs were introduced to the electrospinning solution and a fluorinated acrylic copolymer (FAC) coating was applied, endowing the fabricated membranes with multifunctional properties [102].

Besides the widely used PU matrices, various environmentally friendly matrices have been increasingly investigated in recent years. In one study, Han et al. (2008) fabricated core–shell-structured nanofibers, using polycarbonate (PC) and PU as the shell and the core, respectively. These nanofibers, produced via coaxial electrospinning, could demonstrate an effective performance in various textile applications, such as exposure suits and aviation dresses, exhibiting a satisfactory breathability with a WVTR of 3420 g/m^2^/24 h [7]. In another study, CS was combined with ZnO NPs to create a functionalized electrospun nanoweb, coated on polypropylene non-woven fabric. With an optimum coating density of 0.04 g/m^2^ and average fiber diameter of 700–715 nm, the produced nanoweb performed as a permeable membrane, enabling water vapor and air transmission while impeding the penetration of chemical warfare agents, thanks to its good morphological structure [103].

#### 2.4.2. PVA-Based

In a more recent study by Fadil et al. (2022), an electrospun nanofiber filter (NF) of polyvinyl acid (PVA) was fabricated to enhance the performance properties of protective facial masks. After inserting the produced filter into the facial mask, its filtration efficiency increased. However, the water vapor permeability was reduced to 1440 g/m^2^/24 h due to smaller interspaces in the filter layer, with a further reduction detected after 6 h of use due to the high moisture content, resulting from the filled interfacial gap. The evaluation suggested that the insert did not drastically affect the wearer’s comfort, while the permeability properties could be improved by decreasing the fiber size [87].

#### 2.4.3. PLA-Based

Y. Gu et al. (2022) utilized a PLA matrix to fabricate multifunctional and breathable membranes for personal protective textiles. In this study, eco-friendly PLA was successfully combined with TiO_2_ NPs to fabricate hydrophobic/superhydrophilic Janus membranes that exhibited a greater water vapor permeability on the outer (superhydrophilic) side compared to the inner (hydrophobic) side for the optimum 10 min electrospinning duration. Increasing the electrospinning duration resulted in a thicker PLA nanofiber accumulation that blocked the fibrous membrane’s pores and channels, reducing the water vapor permeability [104].

For the fabrication of breathable membranes with superior water vapor permeability properties, Chen et al. (2023) employed a three-step method. This involved the electrospinning of hydrophilic-oriented PAN (HOPAN) on a PLA nonwoven surface, dip-coating in the JF-8046 hydrophilic agent, and single-side electrospraying a PVDF dilute solution on HOPAN. The result was a hydrophilic-oriented PAN (HOPAN)/hydrophilic PLA @PVDF (HPLA@PVDF) composite membrane with an excellent WVTR of approximately 11,600 g/m^2^/24 h from the HPLA@PVDF to HOPAN side and a slightly reduced but also efficient WVTR of 5200 g/m^2^/24 h from the opposite side. Chen et al. attributed the membrane’s superior breathability to the highly oriented fibers, differential pore size, and asymmetric wettability between the two layers of the fabric [105].

All the works on electrospun films with water vapour breathability are provided in Table 5.

**Table 5:** Bio-based polymers, materials, and electrospinning parameters for the fabrication of fibers with water vapor permeability properties along with their structure and target application.

Matrix	Solvent	Additional Agent	Optimum Process Conditions	Structure	Water Vapor Permeability (Expressed as WVTR)	Target Application	Reference
PU	THF:N,N-dimethylformamide mixture (60:40 *v*/*v*)		13 wt%/v of commercial PU, 10 h of electrospinning, 13 kV voltage, 13 cm tip-to-collector distance, 150 rpm rotating speed, 400 mm/min traverse speed of the drum	Nanofibrous web with a thickness of 252.1 μm and average fiber diameter of 480 nm	40.67 g/m^2^/24 h	Protective clothing	[82]
PU	THF:N,N-dimethylformamide (DMF) (60:40, *v*/*v*)		13% *w*/*w* PU, 2 h electrospinning, 14 kV voltage, 0.6 mL/h feed rate through a 22 G blunt tipped needle, 170 mm adjusting distance between nozzles and collector	Nanofiber layers with an average diameter of fibers 415 nm and a thickness of 0.010 mm	1.72 × 10^3^ g/m^2^/24 h	Various applications (sportswear, protective clothing, orthopedic dressing etc.)	[79]
PU	N,N-dimethylformamide (DMF)		18 wt% PU, 15 kV voltage, 0.6 mL/h feed rate, 20 cm tip-to-collector distance, 300 rpm rotating rate of the collector	Fibrous membranes with fibers’ average diameter of 639 nm	~9.20 × 10^3^ g/m^2^/24 h	Various applications (protective clothing, water purification, and tissue engineering)	[80]
PU	N,N-dimethylacetamide (DMAc)		12% *w*/*w* PU, 13 kV voltage, 10 cm tip-to-collector distance	Fibers with an average diameter of 1.45 μm—PU web/fabric	9.02 × 10^3^ g/m^2^/24 h	Protective and specialty textiles	[76]
PU	N,N-dimethylformamide (DMF)		13 wt% PU, 10 kV voltage, feed rate of 0.2 mL/h, 11 cm tip-to-collector distance	Nanofiber web 2-layer fabric system (PU, densely woven polyester) with a thickness of 0.17 mm	~4.20 × 10^3^ g/m^2^/24 h	Waterproof and breathable materials	[90]
PU	N,N-dimethylformamide (DMF)		10 wt% PU, 25 kV voltage, 0.5 mL/h feed rate, 15 cm tip-to-collector distance, 680 rpm rotating speed	Nanofibrous webs with a thickness of 50 μm and an average fiber diameter of 200 nm	1.09 × 10^3^ g/m^2^/24 h	Various applications	[73]
PU, FPU	DMF	-	4 wt% PU, 0.5 wt% FPU, 20 kV voltage, 2 mL/h feed rate, 15 cm tip-to-collector distance	Microfibrous memrbanes with a thickness of 30 ± 2 μm and an average fiber diameter of 995 nm	9.21 × 10^3^ g/m^2^/24 h	Various applications (protective clothing, bioseparation, membrane distillation, tissue engineering and catalyst carriers, etc.)	[88]
PU, FPU	DMF		20 wt% PU, 2 wt% FPU, 35 kV voltage, 4 mL/h feed rate, ambient temperature 25 °C, 25 cm tip-to-collector distance	Hydrophobic fibrous membranes with a mean pore size of 1.2 μm and pore length of 50 μm	11.90 × 10^3^ g/m^2^/24 h	Protective garments	[91]
PU, FPU	LiCl:DMAc ionic liquid	Lithium chloride (LiCl)	14 wt% PU, 1.75 wt% FPU, 0.006 wt% LiCl, 50 kV voltage, 3 mL/h feed rate, 20 cm tip-to-collector distance	Fibrous membranes with a thickness of 20 ± 2 μm	10.90 × 10^3^ g/m^2^/24 h	Various applications (e.g., protective clothing)	[92]
PU, FPU	DMF:THF (1:1, *w*/*w*)	Carbon nanotubes (CNTs)	1.5 wt% PU/FPU, 1.0 wt% CNTs, 5 kV voltage, 5 mL/h feed rate, 20 cm tip-to-collector distance	Fibrous membranes with a thickeness of 30 ± 2 μm	9.20 × 10^3^ g/m^2^/24 h	Various applications (e.g., protective clothing)	[93]
Ethanol-soluble PU (EPU), FPU	Ethanol	Thymol	14 wt% EPU, 1:8 *w*/*w* FPU/EPU, 8 wt% thymol, 11 kV voltage, 1.5 mL/h feed rate, 12 cm tip-to-collector distance	Nanofibrous membranes with a thickness of 20 ± 2 μm and an average fiber diameter of ~900 nm	3.56 × 10^3^ g/m^2^/24 h	Wound dressings, flexible electronic sensors	[94]
PU	Tetraethoxysilane (TEOS):acetic acid (1:2 *w*/*w*)	Hydrophobic SiO_2_ SNPs	8.2 wt% PU, 5 wt% SNP, 14 kV voltage, 0.2 mL/h feed rate, 18 cm tip-to-collector distance	Superhydrophobic webs with an average fiber diameter of 752 ± 149 nm	6.16 × 10^3^ g/m^2^/24 h	Textile laminate materials	[85]
PU	DMF:BuAc mixture (4:6 *v*/*v*)	Superhydrophobic silica NPs (SSNPs)	18 wt% PU, 9 wt% SSNPs relative to the PU concentration, 12 kV voltage, 0.6 mL/h feed rate, 100 rpm rotating rate	Hydrophobic nanofibrous webs	8.07 × 10^3^ g/m^2^/24 h	Various applications	[95]
PU	Water	Silicon dioxide (SiO_2_), PU emulsion, 4,4′-methylenebis-(phenyl isocyanate) (4,4′-MDI) and 3-aminopropyltriethoxysilane (APTES), triethylamine (TEA, AR) (V_TEOS_:V_EtOH_:V_water_ = 1:4:2)	16% PU emulsion, 0.4 wt% 4,4′-MDI, 2 wt% APTES, 25 kV voltage, 2 mL/min feed rate, 23 cm tip-to-collector	Superhydrophobic membranes with an average fiber diameter of 245 nm	10.12 × 10^3^ g/m^2^/24 h	Waterproof materials	[9]
PU	N,N-dimethylacetamide (DMAc)	PU elastomer (C4FPU), AgNO_3_	2% C4FPU solution, (0.005 wt% AgNO_3_) 45 kV voltage, 2 mL/h feed rate, 22 cm tip-to-collector distance	Fibers with diameter of 460 nm	13.40 × 10^3^ g/m^2^/24 h	Protective textiles	[96]
Waterborne PU (WPU)	Water	Polycarbodiimide (PCD) and long-chain alkyl polymer (LAP) emulsions (PCE and LAE), PEO)	9 wt% PCE, 15 wt% LAE, 0.6 wt% PEO, 40 kV voltage, 4 mL/h feed rate, 22 cm tip-to-collector	Nanofibrous membranes with an average fiber diameter of 548 nm and a thickness of 150 ± 5 μm	4.89 × 10^3^ g/m^2^/24 h	Medical hygiene, wearable electronics, water desalination, and oil/water separation	[8]
Waterborne PU (WPU)	Water	Trimethylolpropane tris (2-methyl-1-aziridine propionate) (TTMA), PEO, Water-based fluoropolymer emulsions (WFE)	35 wt% PU solid contnent in emulsion, 0.6 wt% PEO, 3 wt% TTMA, 6 wt% WFE, 30 kV voltage, 2 mL/h feed rate, 22 cm tip-to-collector, 50 rpm roller speed	Nanofibrous membranes with an average fiber diameter of 680 nm	13 × 10^3^ g/m^2^/24 h	Medical hygiene, wearable electronics and outdoor clothing	[97]
Siliceous PU (SIPU)	DMAC:acetone (2:3 *v*/*v*)	Stearic acid (SA)	11 wt% SIPU, 0 wt% SA, 25.5 kV voltage, 22 cm tip-to-collector	Nanofibrous membranes with a thickness of 60 ± 5 μm and an average fiber diameter of 210 nm)	8.9 × 10^3^ g/m^2^/24 h	Outdoor protective clothing, medical clothing, intelligent clothing and military products	[98]
Silicon-based PU (Si-PU), PMMA	DMAC:acetone (4:6 *v*/*v*)	Octadecanethiol (thiol) and 2, 4, 6, 8-tetramethyl-2, 4, 6, 8-tetra-vinylcyclotetrasiloxane (TMTVSi)	13 wt% Si-PU/PMMA, 20 wt% TMTVSi, 30 wt% thiol, 25 kV voltage, 0.08 mL/min feed rate, 18 cm tip-to-collector	Nanofibrous membranes with an average fiber diameter of ~470 nm	7.88 × 10^3^ g/m^2^/24 h	Protective garments and oil–water separation	[99]
Silicon-based PU (SiPU)	N, N-dimethylacetamide (DMAc):acetone (6:4)	PMMA, Lithium chloride (LiCl)	13 wt% Si-PU and PMMA, 7:3 (*w*/*w*) Si-PU/PMMA, 0.01 wt% LiCl, 25 kV voltage, 0.48 cm h^−1^ flow rate, 18 cm tip-to-collector, 80 °C temperature heat treatment	Nanofiber membrane	9.37 × 10^3^ g/m^2^/24 h	Protective clothing, outdoor equipment, and high-altitude garment	[100]
PU, PAMPS	THF:DMF (Nozzle 1: 60:40, Nozzle 2: 0:100)	GO	Nozzle 1: 6 *w*/*w*% PU, 12 kV voltage, 0.5 mL/h feed rate, 14 cm tip-to-collector distance Nozzle 2: 20 *w*/*w*% PAMPS, 14 kV voltage, 0.1 mL/h feed rate 14 cm tip-to-collector distance, 100 rpm speed of collector, 16 cm/min traverse motion of collector	Nanofibrous membranes	0.86 × 10^3^ g/m^2^/24 h	Protective clothing, wound dressing	[84]
PU, PAMPS	THF:DMF (Nozzle 1: 60:40, Nozzle 2: 0:100)	GO	Nozzle 1: 6 *w*/*v*% PU, 12 kV voltage, 0.5 mL/h feed rate 14 cm tip-to-collector distance Nozzle 2: 30 *w*/*w*% PAMPS, 0.4 %*w*/*v* GO, 14 kV voltage, 0.1 mL/h feed rate, 14 cm tip-to-collector distance 100 rpm speed of collector, 16 cm/min traverse motion of collector	Nanofibrous membranes with mean fiber diameter of 70 nm	~1.43 × 10^3^ g/m^2^/24 h	Protective clothing, wound dressing	[101]
PCL based PU	PCL diol, 4,40-diphenylmethane diisocyanate (MDI), and 1,4-butanediol (BD)		4 wt% PU, 6.5 kV voltage, 1.0 mL/h feed rate, 5–20 cm tip-to-collector distance	Shape memory PU (SMPU) web with a thickness of 40 μm	~5.90 × 10^3^ g/m^2^/24 h	Intelligent clothing material	[81]
PAN, PU	N,N-dimethylacetamide (DMAc)	TiO_2_ NPs, fluorinated acrylic copolymer (FAC)	10 wt% PAN/PU (8/2 mass ratio), 1 wt% TiO_2_ NPs, 2 wt% FAC, 30 kV voltage, 1 mL/h feed rate, 20 cm tip-to-collector distance	Nanofibrous membranes with an average diameter of ~350 nm	12.90 × 10^3^ g/m^2^/24 h	Various applications (high-altitude garments, protective clothing, covering materials, self-cleaning materials, and other medical products etc.)	[102]
PU	DMF:THF mixture (3:2 *v*/*v*)		12% *w*/*w* PU, 12 kV voltage, 0.8 mL/h feed rate, 13 cm tip-to-collector distance, 300 rpm collector speed	Nanofiber webs with fibers’ average diameter of 890 nm	0.79 × 10^3^ g/m^2^/24 h	Army combat, sports uniforms	[77]
PU, C6FPU (20:1)	N,N-dimethylacetamide (DMAc)	Lithium chloride (LiCl)	12 wt% PU, 1.8 wt% C6FPU, 0.004 wt% LiCl, 50 kV voltage, 5 mL/h feed rate, 20 cm tip-to-collector distance, 680 rpm rotating speed	Fibrous membranes with an average fiber diameter of 421 μm and thickness of 19.2 μm	11.80 × 10^3^ g/m^2^/24 h	Protective clothing	[106]
Polycarbonate (PC)	Tetrahydrofuran (THF):dimethylformamide (DMF) (1:1 *v*/*v*)	PU	20 wt% PC, 10 wt% PU, 21 kV voltage, 2.00 mL/h PC feed rate, 1.50 mL/h PU feed rate, 12–13 cm tip-to-collector distance	Nanofibers with a diameter range of 251–1813 nm, specimen thickness 0.240 (0.020) nm	3.42 × 10^3^ g/m^2^/24 h	Textile applications	[7]
CS	Trifluroacetic acid:dichloromethane (2:8 ratios)	ZnO NPs	13% CS, 65 kV voltage, 13.5 cm distance between spinning electrodes, 8 rpm rotation of drum spinning electrode	Nanoweb with an average fiber diameter of 700–715 nm, coating density of 0.04 g m^−2^ and mean pore size of 23.8 μm	0.70 × 10^3^ g/m^2^/24 h	Next-generation lightweight NBC suit for soldiers	[103]
PVA	Water		10 wt% PVA solution, 15 kV voltage, 0.5 mL/h feed rate, 10 cm tip-to-collector	Random orientation network of fibers with diameters of 12–13 μm	1.44 × 10^3^ g/m^2^/24 h	Face mask	[87]
PLA	Dichloromethane (DCM):N,N-Dimethylformamide (DMF) (7:3 wt/wt), ethanol	TiO_2_ NPs	10 wt% PLA, 18 kV voltage, 0.03–0.08 mL/min feed rate, 15 cm tip-to-collector, 10 min	Janus fibrous membrane with an average diameter of about 482 nm	3.03 × 10^3^ g/m^2^/24 h	Multifunctional personal protective materials	[104]
PAN, PVDF	Dimethylformamide (DMF)	Hydrophilic agent JF-8046	HOPAN/HPLA membranes: 4.5 g PAN powder into 45.5 g DMF, 30 kV voltage, 2 mL h^−1^ feed rate, 24 cm tip-to-collector, 80 rpm speed of rotating drum, 10 wt% JF-8046, 2 h electrosprayed PVDF membrane: 2 g PVDF pellets into 48 g DMF, 30 kV voltage, 1 mL h^−1^ feed rate, 10 cm tip-to-collector	HOPAN/HPLA@PVDF membrane with an average fiber diameter of 287 nm	11.6 × 10^3^ g/m^2^/24 h (from HPLA@PVDF to HOPAN side), 5.2 × 10^3^ g/m^2^/24 h (from HOPAN side to HPLA@PVDF)	Quick-drying applications, wicking textiles	[105]

### 2.5. Waterproof Protection

Textile waterproofness is another factor that can be adjusted via the electrospinning method, without water vapor permeability, as mentioned above, being compromised. Waterproof, breathable fabrics are of high interest for protective clothing and sportswear industries, and thus eco-friendly and sustainable matrices have been extensively investigated for their fabrication in recent years. The waterproofness of a fabric is evaluated by its resistance to water penetration, commonly tested through hydrostatic pressure tests. Based on their water resistance values, waterproof fabrics can be categorized into three groups: low-water-resistant (3–8 kPa), middle-water-resistant (10–25 kPa), and high-water-resistant (50–295 kPa) [76]. A surface’s waterproofness can be also determined by the contact angle of droplets, where hydrophobic surfaces typically exceed 90° [79].

#### 2.5.1. PU and FPU-Based

PU webs have been examined for their water-resistant properties, with their fabrication involving the dilution of PU in the electrospinning solution within the optimal concentration range of 10–13 wt%. Several studies report varying water-resistant properties for plain PU webs, ranging from low [77,81,82] to middle [73], attributing lower water resistance to a low thickness or/and the web’s lack of uniformity, due to less controlled manufacturing conditions. In other studies, PU electrospun nanofiber webs were applied on substrate fabrics to examine changes in waterproofness. PU fibers directly electrospun onto polyester/nylon fabric slightly enhanced the water resistance of the fabric system [76], while a three-layered system of PU/polyester/nylon fabric exhibited a superior hydrostatic pressure of approximately 68.6 kPa [90]. In a study by Amini et al. (2016), PU nanofiber layers with thickness of 0.023 mm were examined to assess the system’s wettability and acidic water permeation to protect against acidic rain. The results indicated that the PU layers required 1200 s for acidic water permeation with a 4 h electrospinning duration, while the contact angle was approximately 141.88° for the same duration. By increasing the process duration, the resistance properties were enhanced, as the layer thickness increased [79].

**Figure 5:**
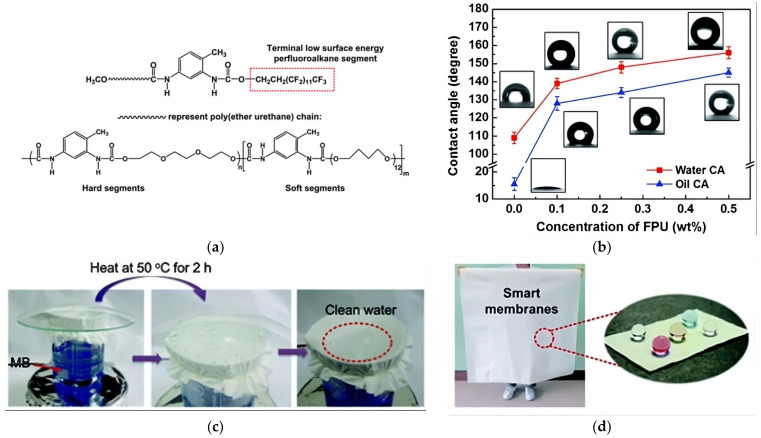
(**a**) Chemical structure of synthesized FPU [83] (reproduced with permission from Wang et al. copyright 2012, Royal Society of Chemistry), (**b**) water contact angles and oil contact angles of PU/FPU membranes (the insets show the corresponding optical profiles of water and oil droplets) [88] (reproduced with permission from Ge et al. copyright 2013, John Wiley and Sons), (**c**) PU/FPU membranes, which can enable water vapor transmission but prevent water from penetrating [88], and (**d**) photograph showing FPU membrane and droplets of water (outside droplets) and oil (centered yellow droplet) on the FPU membrane [83].

With the aim of improving the water-resistant values of PU electrospun nanofibers, the incorporation of FPU into PU matrices has been investigated. FPU is recognized as a hydrophobic agent, associated with lowering the fabric’s surface energy due to its perfluoroalkyl segments, as shown in its chemical structure in Figure 5a [91,93]. As anticipated, electrospun membranes fabricated with a PU/FPU electrospinning solution demonstrated an excellent waterproofness, exhibiting high water-resistant values over 80 kPa [91,92,93,106]. Specifically, Y. Li et al. (2015) generated electrospun membranes with a superior hydrostatic pressure of 108 kPa, using a PU/FPU matrix solution encapsulating carbon nanotubes (CNTs). This addition increased the fabric’s surface roughness, resulting in an apparent rise in the water contact angle and a consequent increase in water-resistant values [93]. Similarly, L. Zhang et al. (2015) fabricated waterproof PU/FPU fibrous membranes and enhanced their water resistance by incorporating lithium chloride (LiCl) into the electrospinning solution. LiCl had a significant impact on the macromolecule’s orientation and porous structure, ultimately leading to a higher hydrostatic pressure of 82.1 kPa for the membranes [92]. FPU containing short perfluorohexyl (-C_6_F_13_) chains has also been combined into a PU solution, yielding membranes with excellent waterproof properties, attributed to the hydrophobicity of fluorinated segments [106]. Additionally, PU/FPU matrices have been employed to produce amphiphobic nanofibrous membranes capable of providing efficient protection against both water and oil, without losing their water vapor permeability properties, as shown in Figure 5c, where the water vapor transmission is enabled at 50 °C, but the water is prevented from penetrating the film (Figure 5c). Actually, by increasing the FPU concentration in the matrix solution, the water contact angle increased from 109° to 156°, while the oil contact angle (OCA) reached 145°, starting from a very low OCA of 15°, as shown in Figure 5b [88]. Furthermore, the encapsulation of SiO_2_ NPs into a polymeric solution of PU/FPU was studied by J. Wang et al. (2012), and it was proved that the properties of the membranes were enhanced, enabling them to load up to 1.5 kg of water and oil while exhibiting superhydrophobicity (WCA of 165°) and superoleophobicity (OCA of 151°) [83] (Figure 5d).

Among the various additional agents encapsulated in plain PU matrices, the combination of a PU elastomer (C4FPU) containing a double-terminal short perfluorobutyl (-C_4_F_9_) chain and AgNO_3_ resulted in electrospun fibers exhibiting a superior hydrostatic pressure of 102.8 kPa. C4FPU endowed the PU/C4FPU/AgNO_3_ membrane with hydrophobicity, owing to its fluorinated segments, while AgNO_3_ significantly enhanced its waterproofness by reducing the maximum pore size (d_max_) of the electrospun nanofibers [96]. SiO_2_ NPs (SNPs) served as another additional agent that increased, to an extent, the hydrophobicity of the membranes, thereby improving their waterproofness. It has been demonstrated that incorporating hydrophobic SNPs results in a rough fiber surface and lower surface energy [9]. When tetraethoxysilane (TEOS) was added in an optimal concentration to the electrospinning solution, SIO_2_ NPs were uniformly dispersed on and in the fibers, further enhancing the hydrophobicity of the membranes. In contrast to C4FPU/AgNO_3_, SNPs/TEOS may enhance membranes’ hydrophobicity but exhibit a lower hydrostatic pressure, leading to low or middle water resistance [85,95]. Similarly, the incorporation of hydrophobic silica gel (HSG) into a PU polymeric solution imparted a hydrophobic surface to the produced fabric, but did not lead to high water-resistance values, as demonstrated by Gu et al. (2018). The fabricated PU/HSG membranes, with an average fiber diameter of 331 nm, exhibited a high water contact angle of approximately 142°, but their hydrostatic pressure value was only 5.45 kPa [80].

Fluorine-free waterborne PU (WPU) membranes, produced through emulsion electrospinning, exhibited an extremely poor hydrostatic pressure due to inadequate hydrophobicity, and thus, various additional agents have been introduced into the electrospinning emulsion to enhance the waterproofness of the membranes. W. Zhou et al. (2021) improved the waterproofness of WPU electrospun membranes by the in-situ dropping of long-chain alkyl polymer and polycarboddimide emulsions (LAE and PCE) into the WPU emulsion. This process was already described in Section 2.4, which involved heating nanofibers at high temperatures to promote the surface migration and enrichment of long-chain alkyls, ensuring robust hydrophobicity by reducing their surface energy. By increasing the LAE concentration to an optimal 15 wt%, while maintaining a constant 9 wt% PCE, the hydrophobicity of the membranes increased, resulting in an effective water contact angle of 137.1° and a high hydrostatic pressure of 35.9 kPa [8]. One year later, W. Zhou et al. (2022) fabricated WPU nanofibrous membranes with even better waterproof properties by incorporating trimethylolpropane tris (2-methyl-1-aziridine propionate) (TTMA), PEO, and water-based fluoropolymer emulsions (WFE) into a WPU matrix at optimum concentrations. Benefiting from the introduction of low-surface-energy fluorinated segments and the rougher structure induced by beaded nanofibers, the membranes exhibited an excellent water resistance with a hydrostatic pressure of 76.9 kPa and a water contact angle of 143.4° [97].

Eco-friendly electrospun nanofibrous membranes with high water-resistant properties have been produced using siliceous PU (SIPU) due to its inherent hydrophobic characteristics. SIPU matrices are typically blended with polymethylmethacrylate (PMMA) and other hydrophobic additional agents to enhance the hydrophobicity and waterproofness of the resulting membrane. It has been observed that an optimal high-temperature post-heat treatment [97] or UV irradiation [99] are necessary after electrospinning to stabilize the membranes’ porous structure and achieve hydrostatic pressure values of up to 65 kPa. In the study of P. Zhang et al. (2022), SIPU nanofibrous membranes were fabricated via electrospinning, demonstrating superior water resistance with a hydrostatic pressure of 87.5 kPa. This notable water-resistant value was attributed to the inclusion of 50 wt% stearic acid, a fatty acid with eighteen alkyl groups that gave hydrophobicity to both the molecule and the produced membranes [98].

The combination of PU with PAN in the matrix solution, along with TiO_2_ NPs as an additional agent, resulted in membranes that also demonstrated waterproof properties. These properties were further enhanced by the addition of a fluorinated acrylic copolymer (FAC) coating, providing superhydrophobic wettability (water contact angle of 152.1°) and enhanced waterproofness (hydrostatic pressure of 62 kPa). Optimizing the FAC concentration to 2 wt%, the low surface hydrophobic agent improved the membrane hydrophobicity by reducing its porosity, while a further increase in the FAC concentration led to a reduction in the hydrostatic pressure (54 kPa) [102].

#### 2.5.2. PVA-Based

Finally, among other eco-friendly polymeric matrices, PVA with encapsulated ZnO NPs and PCL have been utilized to create hydrophobic multifunctional membranes. In a study by Khan et al. (2019), hydrophobicity was imparted to PVA multifunctional membranes by encapsulating ZnO NPs, increasing the water contact angle from 88° (hydrophilic) to 118° (hydrophobic) [44]. In another study conducted by L. Zhou et al. (2021), the hydrophobic characteristics of PVA were utilized to fabricate PVA membranes with a water contact angle of 140.5°, achieved with an optimal concentration of 20 wt% PCL [53].

The waterproof properties of the various bio-based polymer fiber mats that were discussed in this section are summarized in Table 6.

**Table 6:** Bio-based polymers, materials, and electrospinning parameters for the fabrication of fibers with waterproof properties along with their structure and target application.

Matrix	Solvent	Additional Agent	Optimum Process Conditions	Structure	Waterproof Efficacy	Target Application	Reference
PU	N,N-dimethylacetamide (DMAc)		12% *w*/*w* PU, 13 kV voltage, 10 cm tip-to-collector distance	Fibers with an average diameter of 1.45 μm—PU web/fabric	Hydrostatic pressure (3.58 × 10^3^ Pa)	Protective and specialty textiles	[76]
PU	THF:N,N-dimethylformamide (DMF) (60:40 *v*/*v*)		13% *w*/*w* PU, 4 h electrospinning, 14 kV voltage, 0.6 mL/h feed rate, 170 mm tip-to-collector distance	Nanofiber layers with an average fiber diameter of 447 nm and thickness of 0.023 mm	Contact angle (141.882°), Acidic water permeation (>1200 s)	Various applications (sportswear, protective clothing, orthopedic dressing etc.)	[79]
PU	DMF:THF (3:2 *v*/*v*)		12% *w*/*w* PU, 12 kV voltage, 0.8 mL/h feed rate, 13 cm tip-to-collector distance, 300 rpm collector speed	Nanofiber webs with fibers’ average diameter of 890 nm	Resistance to water penetration (4.2 × 10^3^ Pa)	Army combat, sports uniforms	[77]
PU	THF:N,N-imethylformamide (DMF) (60:40 *v*/*v*),		13 wt%/v PU, 13 kV voltage, 130 mm nozzle-to-collector distance, rotational speed of 150 rpm, traverse speed of 400 mm/min, 12 h duration	Nanofibrous web with a thickness of 280.0 μm and average fiber diameter of 480 nm	Hydrostatic pressure (7.35 × 10^3^ Pa)	Protective clothing	[82]
PU	PCL diol, 4,40-Diphenylmethane diisocyanate (MDI), 1,4-butanediol (BD)		4 wt% PU, 6.5 kV voltage, 1.0 mL/h feed rate, 5–20 cm tip-to-collector distance	Shape memory PU (SMPU) web with a thickness of 40 μm	Hydrostatic pressure (5.39 × 10^3^ Pa)	Protective and thermally intelligent clothing material	[81]
PU	N,N-dimethylformamide (DMF)		10 wt% PU, 25 kV voltage, 0.5 mL/h feed rate, 15 cm tip-to-collector distance, 680 rpm rotating speed	Nanofibrous webs with a thickness of 60 μm and an average fiber diameter of 200 nm	Water pressure resistance (15.2 × 10^3^ Pa), water contact angle (130°)	Various applications	[73]
PU	N,N-dimethylformamide (DMF)		13 wt% PU, 10 kV voltage, 0.2 mL/h feed rate, 11 cm tip-to-collector distance	Nanofiber web 3-layer fabric system (PU, polyester fabric, nylon tricot) with a thickness of 0.46 mm	Hydrostatic pressure (~68.6 × 10^3^ Pa)	Waterproof materials	[90]
PU, FPU	N,N-dimethylformamide (DMF)		20 wt% PU, 2 wt% FPU, 35 kV voltage, 4 mL/h feed rate, 25 cm tip-to-collector distance	Hydrophibicfibrous membranes (thickness of 125 μm) and flat films (thickness of 15 ± 1 µm)	Hydrostatic pressure (86 × 10^3^ Pa), water contact angle (149°)	Various applications (e.g., protective clothing)	[91]
PU, FPU	DMF:THF (1:1 *w*/*w*),	Carbon nanotubes (CNTs)	1.5 wt% PU/FPU, 0.75 wt% CNTs, 5 kV voltage, 5 mL/h feed rate, 20 cm tip-to-collector distance	Fibrous membranes with a thickness of 30 ± 2 μm	Hydrostatic pressure (108 × 10^3^ Pa)	Various applications (e.g., protective clothing)	[93]
PU, FPU	LiCl, DMAc ionic liquid	Lithium chloride (LiCl)	14 wt% PU, 1.75 wt% FPU, 0.006 wt% LiCl, 50 kV voltage, 3 mL/h feed rate, 20 cm tip-to-collector distance	Fibrous membranes with a thickness of 20 ± 2 μm	Hydrostatic pressure (82.1 × 10^3^ Pa)	Various applications (e.g., protective clothing)	[92]
PU, C6FPU	N,N-dimethylacetamide (DMAc)	Lithium chloride (LiCl)	12 wt% PU, 1.8 wt% C6FPU, 0.004 wt% LiCl, 50 kV voltage, 5 mL/h feed rate, 20 cm tip-to-collector distance, 680 rpm rotating speed	Fibrous membranes with an average fiber diameter of 421 μm and thickness of 19.2 μm	Hydrostatic pressure (88.2 × 10^3^ Pa), water contact angle (142.6°)	Protective clothing	[106]
PU, FPU	DMF	-	4 wt% PU, 0.5 wt% FPU, 20 kV voltage, 2 mL/h feed rate, 15 cm tip-to-collector distance	Microfibrous membranes with a thickness of 30 ± 2 μm and an average fiber diameter of 995 nm	Hydrophobicity (water contact angle of 156°) Oleophobicity (oil contact angle of 145°)	Protective clothing, bioseparation, membrane distillation, tissue engineering	[88]
PU	Water	Silicon dioxide (SiO_2_), PU emulsion, 4,4′-methylenebis-(phenyl isocyanate) (4,4′-MDI) and 3-aminopropyltriethoxysilane (APTES), triethylamine (TEA, AR) (V_TEOS_:V_EtOH_:V_water_ = 1:4:2)	16% PU emulsion, 0.4 wt% 4,4′-MDI, 2 wt% APTES, 25 kV voltage, 2 mL/min feed rate, 23 cm tip-to-collector	Superhydrophobic membranes with an average fiber diameter of 245 nm	Hydrostatic pressure (8.02 × 10^3^ Pa), water contact angle (154°)	Waterproof materials	[9]
PU	Tetraethoxysilane (TEOS):acetic acid (1:2 *w*/*w*)	Hydrophobic SiO_2_ SNPs	8.2 wt% PU, 5 wt% SNP, 14 kV voltage, 0.2 mL/h feed rate, 18 cm tip-to-collector distance	Superhydrophobic webs	Static contact angle (151.3 ± 5.9), shedding angle (32.6 ± 1.7)	Textile laminate materials	[85]
PU	DMF:BuAc (4:6 *v*/*v*)	Superhydrophobic silica NPs (SSNPs), Tetraethoxysilane (TEOS)	18 wt% PU, 9 wt% SSNPs relative to the PU concentration, 6 wt% TEOS, 12 kV voltage, 0.6 mL/h feed rate, 100 rpm rotating rate	Hydrophobic nanofibrous webs	Hydrostatic pressure (23.5 × 10^3^ Pa), water contact angle (139°)	Various applications	[95]
PU	N,N-dimethylformamide (DMF)	Hydrophobic silica gel (HSG)	18 wt% PU/HSG, 3 wt% HSG with respect to the polymer PU, 15 kV voltage, 0.6 mL/h feed rate, 20 cm tip-to-collector distance, 300 rpm rotating rate of the collector	Fibrous membranes with an average diameter of 331 nm	Hydrostatic pressure (5.45 × 10^3^ Pa), contact angle (~142°)	Various applications (protective clothing, water purification, and tissue engineering)	[80]
Waterborne PU (WPU)	Water	Polycarbodiimide (PCD) and long-chain alkyl polymer (LAP) emulsions (PCE and LAE), PEO	9 wt% PCE, 15 wt% LAE, 40 kV voltage, 4 mL/h feed rate, 22 cm tip-to-collector	Fluorine-free nanofibrous membranes with an average fiber diameter of 548 nm and thickness of 150 ± 5 μm	Hydrostatic pressure (35.9 × 10^3^ Pa), water contact angle (137.1°)	Green and high-performance fibrous materials used for medical hygiene, wearable electronics, water desalination, and oil/water separation	[8]
Waterborne PU (WPU)	Water	Trimethylolpropane tris (2-methyl-1-aziridine propionate) (TTMA), PEO, Water-based fluoropolymer emulsions (WPE)	35 wt% PU solid content in emulsion, 0.6 wt% PEO, 3 wt% TTMA, 22 wt% WPE, 30 kV voltage, 2 mL/h feed rate, 22 cm tip-to-collector, 50 rpm roller speed	Nanofibrous membranes with an average fiber diameter of 680 nm	Hydrostatic pressure (76.9 × 10^3^ Pa), water contact angle (143.4°)	Medical hygiene, wearable electronics, and outdoor clothing	[97]
Silicon-based PU (SiPU), PMMA	DMAC:acetone (4:6 *v*/*v*)	Octadecanethiol (thiol), 2, 4, 6, 8-tetramethyl-2, 4, 6, 8-tetra-vinylcyclotetrasiloxane (TMTVSi)	13 wt% Si-PU/PMMA, 20 wt% TMTVSi, 30 wt% thiol, 25 kV voltage, 0.08 mL/min feed rate, 18 cm tip-to-collector	Nanofibrous membranes with an average fiber diameter of ~470 nm	Hydrostatic pressure (64.43 × 10^3^ Pa), Water contact angle (131°)	Protective garments and oil–water separation	[99]
Siliceous PU (SIPU)	DMAC:acetone (2:3 *v*/*v*)	Stearic acid (SA)	11 wt% SIPU, 50 wt% SA, 25.5 kV voltage, 22 cm tip-to-collector	Nanofibrous membranes with a thickness of 60 ± 5 μm and average fiber diameter of 390 nm)	Hydrostatic pressure (87.5 × 10^3^ Pa), water contact angle (~133°)	Outdoor protective clothing, medical clothing, intelligent clothing, and military products	[98]
PU, PAN	N,N-dimethylacetamide (DMAc)	TiO_2_ NPs, fluorinated acrylic copolymer (FAC)	10 wt% PAN/PU (8/2 mass ratio), 1 wt% TiO_2_ NPs, 2 wt% FAC, 30 kV voltage, 1 mL/h feed rate, 20 cm tip-to-collector distance	Nanofibrous membranes with an average diameter of ~350 nm	Hydrostatic pressure (62 × 10^3^ Pa), water contact angle (152.1°)	High-altitude garments, protective clothing, covering materials, self-cleaning materials, and other medical products, etc.	[102]
FPU	DMF:THF (1:2 *w*/*w*)	SiO_2_ NPs	FPU 18 wt%, SiO_2_ 1 wt%, 18 kV voltage, 0.5 mL/h feed rate, 15 cm tip-to-collector distance	Superamphiphobic nanofibrous membranes with an average fiber diameter of 915 nm and thickness of 50 μm	The membranes could load 1.5 kg water and oil (olive oil) superhydrophobicity (water contact angle of 165°) and superoleophobicity (oil contact angle of 151°)	Protective clothing, bioseparation, water purification, tissue engineering, microfluidic systems, etc.	[83]
PU	N,N-dimethylacetamide (DMAc)	PU elastomer (C4FPU), AgNO_3_	2% C4FPU solution, 0.015 wt% AgNO_3_, 45 kV voltage, 2 mL/h feed rate, 22 cm tip-to-collector distance	Fibers with a diameter of 641 nm	Hydrostatic pressure (102.8 × 10^3^ Pa)	Protective textiles	[96]
Ethanol-soluble PU (EPU), FPU	Ethanol		8 wt% FPU, 1:8 *w*/*w* FPU/EPU, 11 kV voltage, 1.5 mL/h feed rate, 12 cm tip-to-collector distance	Nanofibrous membranes with a thickness of 20 ± 2 μm and an average fiber diameter of 249 nm	Hydrostatic pressure (4.95 × 10^3^ Pa), water contact angle (~144°)	Wound dressings, flexible electronic sensors	[94]
Silicon-based PU (SiPU)	N, N-dimethylacetamide (DMAc):acetone (6:4),	PMMA, Polyvinyl butyral (PVB), Lithium chloride (LiCl)	13 wt% Si-PU and PMMA, 7:3 (*w*/*w*) Si-PU/PMMA, 0.01 wt% LiCl, 40 wt% PVB, 25 kV voltage, 0.48 cm h^−1^ feed rate, 18 cm tip-to-collector, 80 °C temperature heat treatment	Nanofiber membrane	Hydrostatic pressure (65.29 × 10^3^ Pa), water contact angle (139°)	Protective clothing, outdoor equipment, and high-altitude garment	[100]
PVA	Water	Zinc oxide (ZnO) NPs	10% by weight PVA, 9% by weight ZnO, 14 kV voltage, 12 cm tip-to-collector	Nanofibers	Water contact angle (118°)	Medical surgeon	[44]
PCL	Formic acid:acetic acid (7:3 *v*/*v*)		20 wt% PCL, 24 kV voltage, 0.77 mL/h feed rate, 15 cm tip-to-collector	Nanofibers with a diameter of 119.1 ± 24.6 nm	Water contact angle (140.5°)	Health products (wound healing, wound dressings for burn injuries)	[53]

### 2.6. UV-Protection

As the risk of UV radiation is rapidly increasing in recent years due to the stratospheric ozone decrease and the ongoing climate crisis, more individuals are becoming concerned about its hazards and seeking ways to be protected from its unwanted effects. Consequently, textiles and clothing with UV protective properties have garnered the interest of researchers and industries, as they pose ideal materials for personal protective equipment, outdoor/sports, and military textiles [40,47,107].

There are two types of UV radiation that manage to reach the Earth’s surface, overcoming both stratospheric ozone and the atmosphere: UV-A, long-wave UV light that cannot be absorbed by the stratospheric ozone, and UV-B, short-wave UV light that can be partially absorbed by the atmospheric ozone. While small amounts of UV radiation are essential for various vital functions of the body, such as vitamin D synthesis and immune system enhancement, extended direct exposure to the sun has been shown to be associated with skin cancer, premature skin aging, the suppression of the immune system, and cataracts [108,109]. Therefore, the use of UV protective clothing with a high ultraviolet protection factor (UPF), which blocks UV radiation and minimizes the UV-A and UV-B transmittance, is now a matter of health. The Ultraviolet Protection Factor (UPF) is typically evaluated according to the American Association of Textile Chemists and Colorists (AATCC) Test Method 183–2004 Transmittance or Blocking of Erythemally Weighted Ultraviolet Radiation through Fabrics.

#### 2.6.1. PVA-Based

To date, numerous studies have focused on the fabrication of UV protective textiles via electrospinning using various green matrices, including widely used PVA and PU. Starting with a study by K. Lee and Lee (2012), they fabricated a layered fabric system utilizing PVA as a matrix and enhanced its initial UV protection properties by incorporating TiO_2_ NPs into the polymeric solution and simultaneously increasing its web area density. Initially, the addition of 2 wt% TiO_2_ NPs partially increased the ultraviolet protection from UPF = 0 to UPF = 13. Subsequently, when the web area density doubled to 3.0 g m^−2^, UPF rose to 50+ with only 8.8% of UV-A and 0.3% of UV-B light able to transmit to the fabric (K. Lee & Lee, 2. Similarly, Khan et al. (2019) added 9 wt% zinc oxide (ZnO) NPs in a PVA polymeric solution and fabricated multifunctional PVA/ZnO nanofiber composite membranes that exhibited nearly 0% UV transmission [44]. ZnO NPs strongly absorbed in the UV-A and UV-B region, thus contributing to the UV-shielding properties of the membranes. PVA has also been combined with lignin for the fabrication of electrospun nanocomposite fibers with excellent UV protective properties, exhibiting a UPF of 50+ and UV-A and UV-B transmittance of 0.1% [110].

#### 2.6.2. PU-Based

Similarly, PU matrices are usually combined with TiO_2_/ZnO/CeO_2_ NPs or other additional agents to produce functional textiles with UV protective properties and are also modified to enhance these properties [40]. Xu et al. (2017) fabricated electrospun PU/PAN/TiO_2_ nanofibrous membranes with only a 1 wt% concentration of TiO_2_ NPs in the polymeric system solution, and significantly enhanced the membranes’ multifunctionality with a two-step coating modification using 2-hydroxy-4-n-octoxybenzophenone (UV531) and fluorinated acrylic copolymer (FAC). Due to the UV-absorbing properties of UV531, the membranes’ UPF increased from 17 to 1485, demonstrating excellent UV-blocking properties for both UV-A and UV-B light. FAC served only as a hydrophobic agent and adhesive material [102]. In a more recent study, Viscusi et al. (2023) modified PU electrospun membranes utilizing a combination of copper (Cu) and zinc (Zn) oxides with a mol ratio of 1:1 Cu/Zn, and fabricated an excellent UV-protective material with a UPF of 50+. The strong UV-absorption properties of both oxides led to 99.99% blocking UV-A and 100% blocking UV-B capabilities of the membranes. The PU membranes also exhibited excellent UV protective properties when modified with only one of the two oxides [40].

#### 2.6.3. Other Polymers

Natural cotton cellulose is another biodegradable matrix, which requires surface modification or combination with an appropriate additional agent in order to produce functional fibers. In a study by Li et al., electrospun nanofibers of natural cotton cellulose were modified, utilizing a rare-earth nano-oxide material made of cerium dioxide (CeO_2_). After a hydrothermal reaction, the CeO_2_ NPs were well dispersed on the cotton cellulose nanofibers’ surface, significantly enhancing its UV absorption performance due to the large refractive index of the CeO_2_ NPs and their sensitivity to UV light absorption [107].

Eco-friendly electrospun Janus membranes with UV-blocking properties were recently fabricated by Gu et al. (2022), who utilized PLA as a matrix combined with TiO_2_ NPs. The produced fabric exhibited stronger UV-blocking properties than the pure PLA fabric, but still did not exhibit impressive results, blocking only 84–86% of UV-B light [104]. The UV protection properties of the electrospun fiber structures are shown in the next table.

The above-mentioned electrospun fiber mats that provide UV protection are summarized in Table 7. 

### 2.7. Thermal Prοtection

Textiles with thermal properties, like flame retardancy and thermal regulation, are attracting extensive attention and are applied not only in personal protection items when working in environments with high heat radiation, such as firefighting, the military, and industrial protective clothing, but also in the civilian market. Thermal regulation textiles can adjust the microclimate near human skin and provide comfort under a thermal environment, while fire-retardant textiles with a limiting oxygen index value greater than the environment oxygen concentration (21%) can burn at a slower rate, thus offering safety to wearers [38,111,112]. The growing attention paid towards the development of polymer materials that are used in heat-resistant and flame-retardant textiles is evident, and a huge effort has been undertaken to synthesize new textiles with improved properties [111].

Electrospinning is carried out for designing polymers with thermal properties by incorporating phase-changing materials (PCMs). PCMs can absorb and store energy in the form of latent heat with a limited temperature range, increasing the energy storage density and controlling the body temperature [113]. However, conventional PCM-based textiles fabricated by a simple mix of PCMs with textiles have limited practical applications due to some intrinsic weaknesses, which include density changes, phase segregation, low thermal conductivity, and leaking. These issues are solved by coaxial electrospinning, which manufactures fibers with a concentrically aligned ‘core–shell’ structure and effectively encapsulates PCMs inside green polymer ‘shells’ [17,112].

#### 2.7.1. Thermal Regulation

Among the various PCMs, PEG has gained the most interest because of its excellent properties, which include a broad melting point range, high heat enthalpy, consistent melting behavior, and lack of corrosiveness. However, neat PEG has a low molecular weight and viscosity, and it is not appropriate as a matrix for electrospinning [113]. Therefore, it is combined in several studies with other green polymers, such as CA, PVA, PU, and PCL, which act as supporting shells to develop phase-change fibers with an increased encapsulation efficiency [112,113,114,115,116]. Simple and coaxial electrospinning were tested as processes for the production of membranes with thermal regulation capabilities, and the conclusion in all the cases was the same: an increase in PEG content, until a certain value where no encapsulation problems are shown, had a positive impact on the thermal energy storage ability of the fibers, reaching heat enthalpies ranging from 39.5 × 10^3^ J/kg [116] to 78.10 × 10^3^ J/kg [113], as analytically shown in Table 8. PCMs were also studied in the work of Quan et al. (2022), who performed emulsion electrospinning utilizing the PCM dodecanol (DD) and PVA as the oil phase and water phase, respectively, to prepare core-sheath-structured nanofibers with temperature regulation properties and a good thermal cycle stability [117]. Additionally, stearic acid (SA) was examined as a PCM by Zhang et al. (2022), who investigated the thermal regulation of a multifunctional nanofiber mat from hydrophobic siliceous PU (SIPU): PU with SA. The addition of 30 wt% SA improved not only the waterproof performance, but also the thermal regulation performance of the membranes, enabling them to be potentially applied in outdoor protective, medical, and military clothing [98].

**Figure 6:**
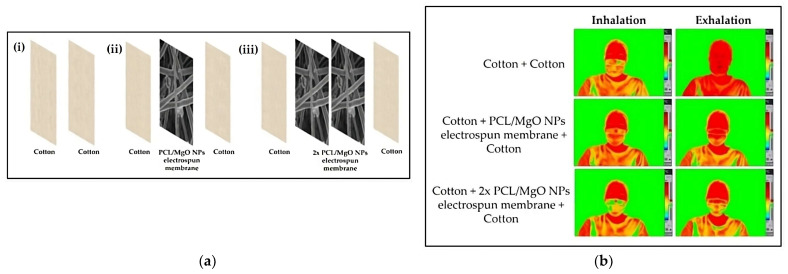
(**a**) Schematic representation of the three configurations evaluated: (**i**) 2 cotton layers; (**ii**) 2 cotton layers with PCL/MgO NPs electrospun membrane; (**iii**) 2 cotton layers with 2 PCL/MgO NPs electrospun membrane; and (**b**) facial temperature distribution for each configuration [52].

Other polymers that have been used for the production of biodegradable polymers with thermal protection for various uses, such as facemasks, include PVA and PCL. Hang et al. first produced melamine/PVA fibers by reaction electrospinning, which have a great potential application in high-temperature-resistant filters thanks to their high decomposed temperature up to 370 °C [118]. As already described in Section 2.2 and Section 2.3, Costa et al. (2021) developed membranes for filter layers of facemasks from PCL and cotton by using electrospinning and functionalizing them with NPs, like Ag, TiO_2_, and MgO (Figure 6a). The aim of this study was to improve the filtration efficiency while including antimicrobial properties and ensuring that the face temperature when wearing the facemask is not increased, leading to overall discomfort. With this aim, facial temperature changes were recorded in the nose, mouth, and cheek and it was proven that the facemasks with MgO accomplished the best facial temperature distribution, with 34.1 °C for nose, 33.5 °C for mouth, and 30.6 °C for cheek, as shown in Figure 6b [52].

#### 2.7.2. Flame Retardancy

Finally, flame-resistant and fire-retardant fabrics for applications mainly in protective military, firefighting, and industrial clothing have become a major social concern, and electrospinning has recently been undertaken when designing this type of textiles using biodegradable polymers [38,111]. Specifically, Mamtha et al. (2023) encapsulated MgO and Ag NPs in PU and investigated their effect on the flame retardancy of the produced polymers. PU:MgO electrospun fibers had the best flame-retardant characteristics (26.51 s after-flame time and 7.67 s after-glow time), since Mg^+^ ions acted as catalysts in cross-linking and dehydration reactions, affecting the char formation, and MgO had the ability to block the melt dripping and flame growth. This sample belonged to the V1 category, which means that the flame extinguished within 30 s with no dripping [38]. Furthermore, Hao et al. (2023) tested the flame retardance properties of PLA fabrics co-electrospun with DiDOPO, a derivative of 6H-dibenz(C,E) (1,2)oxaphosphorin-6-oxide), in order to enhance the poor flame retardation of pure PLA. Actually, the addition of DIPOPO improved the limited oxygen index, shortened the time span of total release by 83%, and led to the V0 category, meaning that the flame extinguished within 10 s with no dripping [68]. 

The above-mentioned the electrospun films that provide thermal regulation are shown in Table 8.

### 2.8. Chemical Protection

The production of chemical protective textiles via electrospinning utilizing green polymers and other eco-friendly raw materials as matrices has been of great interest to researchers and industries over the last decades. Green matrices offer not only sustainability, but also demonstrate a great efficiency in chemical protection when combined with a suitable additional agent. These textiles are used primarily for personal protective equipment (PPE), military purposes, and medical/pharmaceutical industries, as they efficiently prevent hazardous chemicals from coming into contact with the skin, exhibit antioxidant activity, or display decomposition ability towards specific chemicals [119].

Different matrix–additional agent systems exhibit varying chemical protection properties towards one or more chemicals simultaneously. In a study by Lee and Lee (2012), a layered fabric system of TiO_2_ nanocomposite fibers was fabricated, which demonstrated a great formaldehyde decomposition efficiency. The researchers utilized PVA as a matrix and TiO_2_ NPs as the additional agent. The electrospun PVA/TiO_2_ fibers underwent initial heat treatment to stabilize their structure against dissolution in water, and then UV irradiation was conducted to enhance their decomposition efficiency. After a determined optimum 15 h of UV irradiation, nanocomposite fiber webs exhibited a formaldehyde decomposition efficiency of 80%, reducing the formaldehyde gas concentration from 25 to 5 ppm. This same system also provided additional chemical protection against ammonia gas, a highly irritating gas with a suffocating odor. Specifically, after 2 h of UV irradiation, the PVA/TiO_2_ electrospun fibers exhibited an ammonia deodorization efficiency of 32.2% [47].

In another study, Sinha and Das (2018) encapsulated ZnO NPs in CH, a natural biodegradable polysaccharide, in order to fabricate an electrospun nanoweb with chemical protection properties. With an optimum coating density of 0.43 g m^−2^ and the presence of ZnO NPs, the chemical protection increased and the nanofibrous web exhibited a dichloropropane (DCP) penetration time of 270 s. Empirically, this is equal to a 4 h chemical protection, making the web ideal for next-generation military suits [103].

A few years later, Li and Yang (2020) fabricated a rutin-loaded CA/PEO fiber membrane via electrospinning, which provided remarkable antioxidant activity without compromising its superior mechanical properties. The research revealed that even a slight presence of rutin can lead to an incredible increase in the membrane’s antioxidant activity, soaring from 3.3% for the rutin-unloaded membrane to 98.3% for the 2% rutin-loaded membrane. These results render rutin-loaded CA/PEO fiber membranes as ideal candidates for bioactive materials in pharmaceutical or medical industries [56]. The exact materials and optimum processing conditions for the production of all the above-mentioned fiber mats with chemical properties are summarized in Table 9.

### 2.9. Shape Memory

Shape-memory polymers (SMPs) represent smart materials capable of recovering their original shape from a pre-programmed temporary shape when exposed to a range of exterior stimuli, such as a change in the surrounding temperature, pH, UV light magnetic fields and electric fields, solvents, pressure, and humidity [81,120]. SMPs present several advantages, including being lightweight, low-cost, and having easy processing. In particular, SMPs with fibrous structures are attracting attention in applications involving contact with the human body, like smart textiles. Because of its high porosity and surface area, the electrospinning technique has been acknowledged as one of the most appealing and widely employed technologies for the fabrication of this type of smart fabrics using biphasic bio-based polymers [121]. The one-polymer phase provides a definitive shape and the other offers a switching ability by thermal transitions (T_g_ or T_m_) or reversible chemical reactions [122].

#### 2.9.1. PU-Based

PCL-based PU and PU:PEG matrices are capable of forming fiber mats with great shape recovery (R_rec_), fixity (R_f_), and retention rates (R_ret_) via electrospinning. Several works focus on the examination of various solutions and processing parameters during electrospinning as factors that influence the quality and final properties of shape-memory fibers. Starting with Zhuo et al. (2008), the effect of the matrix concentration and the voltage on the diameter of the produced shape-memory PU (SMPU) electrospun fibers was studied and it was concluded that, when adjusting these factors (as seen in Table 10), a high R_rec_ and R_f_ of 98% and 80% were accomplished [123]. Similarly, Chung et al. (2011) reported that the factors that most influence the R_ret_ and R_rec_ of PCL-based SMPU mats are the ratios between the molecular weight and the reactants of the PCL, which result in different soft and hard segment contents. In fact, the study led to the conclusion that the R_ret_ was greater when a high soft segment content was achieved in contrast to R_rec_, which was enhanced when the hard segment content was high [81]. On the same page, Feng et al. (2021) studied different ratios of PU and PEG matrixes in their solution, combining the extreme rigidity of PEG and high elasticity of pure PU. The fabricated PU:PEG electrospun membranes were rigid below the melting point of PEG (Tm ≈ 60 °C) and flexible above it. Actually, when deforming them at T = 20 °C and 60 °C, they did not restore their original shape at the low temperature, whereas when heated, they showed full shape recovery at only 23 s. In addition, the time required for the shape recovery was related to the PEG content of the membranes, and it was noticed that an increase in the PEG content of the matrix extended the needed recovery time [112].

#### 2.9.2. PCL-Based

Another biodegradable electrospun fabric with shape memory properties was constructed by Matsumoto et al. (2012) using a multiblock copolymer (PDLCL) consisting of crystallizable poly(x-pentadecalactone) hard segments (PPDL) and PCL switching segments. In this work, various deformation percentages, specifically 25% and 25–50%, were applied in the electrospun PDLCL fabric, and it was proven that they exhibited a great R_rec_ and R_f_ for a narrow temperature range (T = 40–60 °C) and a small deformation percentage (25%), while R_rec_ was evidently decreased for high deformation percentages [124].

In terms of great biodegradability and shape memory properties, PCL blends are widely used to produce shape-memory structures, since they show rapid shape memory recovery behavior thanks to the melting/recrystallization of PCL as a switching transition. A PCL matrix is frequently electrospun in combination with epoxy composites that provide great strength and deformation; however, they cannot be electrospun without the use of another matrix due to their low molecular weight [125,126]. Zhang et al. (2015) investigated the shape memory behavior of a PCL:epoxy composite electrospun mat that exhibited full shape memory recovery at a temperature higher than the T_m_ of PCL (Tm ≈ 60 °C) [125]. The introduction of iodonium salts, employed as a photo-initiator, into a PCL/epoxy electrospinning solution was studied by Iregui et al. (2017). Iodonium salts played a crucial role by contributing to the polymerization of the epoxy resin, diglycidyl ether of bisphenol A (DGEBA), through cationic photopolymerization. This process mitigated the need for high temperatures that could induce PCL melting. Additionally, iodonium salts were observed to improve spinnability through increased conductivity, resulting in the formation of more uniform fibers with a reduced diameter. The fabrication of electrospun fiber mats with shape memory effects involved a two-step process that included the electrospinning and the photopolymerization of the epoxy monomer post UV curing, which provided the mats with thermal and structural stability. The produced nanofibrous mats exhibited an R_rec_ of 88–100% and R_f_ of 95–99% over five consecutive cycles, while maintaining a stable morphological structure at temperatures exceeding the PCL melting [126]. A few years later, Iregui et al. (2017) evaluated the impact of PCL concentration in the same electrospinning system. They observed that, by increasing the PCL concentration, the recovery ratios were slightly enhanced, leading to R_rec_ > 95%, while an increased applied voltage and flow rate were essential due to the elevated viscosity [122].

#### 2.9.3. PLA-Based

Another environmentally friendly polymer utilized for the fabrication of shape memory fibers is PLA, which exhibits a thermo-responsive shape memory effect when heating up to a switching temperature (T_sw_) higher than its T_g_. At this point, polymer chains have sufficient mobility to recover the original form from a previously fixed temporary shape. In order to bring the T_sw_ closer to the temperature of the human body, Leonés et al. (2019) introduced polylactic acid oligomer (OLA) into the electrospinning solution and evaluated the effect of the PLA/OLA mass ratio on the shape memory behavior at different temperatures. It was reported that the addition of OLA decreased the T_g_ from 60° to a more desired temperature range of 47–24 °C, closer to body temperature, while an increase in the OLA concentration led to less uniform fibers with a lower diameter. At the optimal ratio of 80:20 PLA/OLA, fibrous mats exhibited an R_rec_ equal to 100% at 45 °C and >79% at 40 °C, along with an R_f_ of >95% and 98% at 45 and 40 °C, respectively, resulting in a very promising system for biomedical applications [120]. 

The shape memory features of the various bio-based electrospun fibers that were discussed in this Section are summarized in Table 10.

### 2.10. E-Textiles and E-Skins

In recent times, there has been a remarkable surge in the market of wearable electronic devices integrated with clothing and even skin sensors to enhance human life, health monitoring, medical diagnosis, or entertainment. Nevertheless, a shared technical challenge faced by these devices is their dependence on rechargeable batteries. Consequently, there is a pressing need to address this crucial issue with flexible and sustainable energy sources that can scavenge body motion energy [127]. Regarding e-skins, they should be able to monitor pressure, strain, flexion, movement, and deformation, etc., by converting these physical deformations into electrical signals, while possessing features, such as flexibility and durability, as well as biocompatibility and breathability, most of the time. On the basis of their sensing mechanisms, sensors can be categorized into capacitive, piezoresistive, piezoelectric, and triboelectric sensors [128]. Electrospun nanofibers are employed as templates for absorbing conductive nanomaterials, such as silver, graphene, and carbon or carbon-black-forming structures ranging from three-dimensional e-skins [128] to nanofiber-based sensors in the form of yarns [129].

Although the fields of e-textiles and e-skins have seen tremendous advancements, it is important to recognize that a large percentage of these developments rely on non-bio-based polymers. While synthetic materials contribute to the technological progress in the field, concerns about their environmental impact and sustainability may limit their use in the future. Therefore, over the past five years, there has been a significant effort towards incorporating bio-based polymers into these technologies, highlighting how crucial it is to switch to more environmentally friendly and sustainable alternatives.

#### 2.10.1. PU-Based

In general, a process sequence that is frequently employed for the fabrication of flexible configurations for body motion monitoring is the development of PU/TPU nanofiber mats through electrospinning and their coating through various techniques with conductive materials, such as conducting polymers [130], reduced-GO (RGO) [131], and carbon nanofibers (CNFs) [132] and nanotubes (NTs) [133]. PU and TPU electrospun substrates can offer not only an excellent flexibility, which is crucial where the attachment of the produced arrays on clothes and skin is concerned, but also a large specific surface area and high porosity.

For example, Ding et al. (2017) fabricated highly flexible and stretchable conductive nonwovens by dip-coating electrospun PU fibers in the conducting polymer of poly(3,4-ethylenedioxythiophene):poly(styrenesulfonate) (PEDOT:PSS). The produced array was used as a conductor in an electric circuit to light an LED, and when it was stretched, twisted, or bent, the circuit showed a stable performance, proving their potential to be used as stretchable conductors in wearable electronics [130]. In addition, one year later, Wang et al. (2018) proposed a resistive-type strain sensor able to detect various human motions by forming TPU electrospun mats and immersing them in RGO. The sensors possessed not only a good flexibility thanks to the substrate, but most importantly, satisfactory sensing features, such as sensitivity (11 Gauge factor—GF—in strain of 10% and 79 in strain of 100% in reversible strain regime), stability (stretch/release test of 6000 cycles), and response time (200 ms) when attached on skin or clothes to monitor different human motions [131]. Furthermore, another stretchable strain sensor was produced by the spray coating of CNFs on an electrospun TPU substrate and showed a high sensitivity (17.3 GF in strain of 0–80%) and remarkable durability, too [132]. Nonetheless, the best sensing performance achieved through this type of configuration is that fabricated most recently by Wang et al. (2023) through the dip coating of an electrospun TPU fibrous film in ultrasonication-treated CNTs. This sensor showed a high sensitivity with a maximum GF of 1571, remarkable tensile strength, and wide stretching range (0–400%) [133].

**Figure 7:**
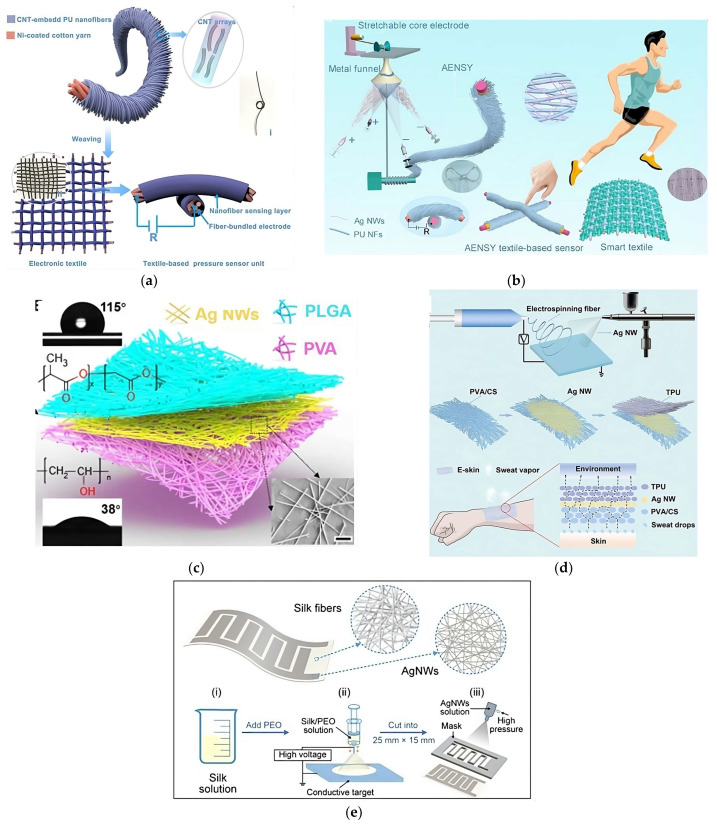
Schematic illustration of: (**a**) the pressure sensor made from CNT-embedded PU fibers and Ni-coated cotton yarn [135] (reproduced with permission from Qi et al. copyright 2020, Elsevier), (**b**) the AENSY textile-based sensor from PU NFs and Ag NWs [136] (reproduced with permission from Dai et al. copyright 2023, ACS Publications), (**c**) the three-dimensional TENG–based e-skin along with images of the water contact angle and molecular structure of PLGA and PVA [128] (reproduced with permission from Peng et al. copyright 2020, American Association for the Advancement of Science), (**d**) the three-layered e-skin composed of a PVA/CS bottom layer, Ag NWs middle layer, and TPU top layer [140] (reproduced with permission from Shi et al. copyright 2021, ACS Publications), and (**e**) the silk fibroin-based sensor composed of the silk fiber film and the interdigital electrode made of AgNWs [129] (reproduced with permission from Wen et al. copyright 2022, Springer Nature).

Other interesting e-fabric arrays that are worth mentioning are formed by coating PU nanofibers doped by either GO [134] or CNTs [135] onto the surface of a Ni-coated cotton yarn and weaving them in order to develop electronic fabrics (Figure 7a). In both cases, the porous nanofiber structures improved the sensitivity of the e-fabrics. More specifically, in the first case, the sensor was able to sense the proximity of a finger within 10 cm, whereas, in the second case, the textile sensor was capable of the real-time monitoring of frowning and relaxation movements of a human forehead. Therefore, they were able to map and quantify mechanical stress and make possible the design of innovative smart textiles for personalized healthcare in the future [134,135].

More recently, another similar, but more complicated weavable Ag NW-embedded PU fiber sensing yarn (AENSY) with a hierarchical architecture was developed through a conjugate electrospraying and electrospinning technique. The stretchable and breathable AENSY textile-based sensor, which is demonstrated in Figure 7b, showed a satisfactory sensitivity (GF 55−1010) and durability and possessed the ability to monitor human movements, ranging from wrist bending and muscle movements to micro-expressions and chewing, in real time. Therefore, this novel method (Figure 7b) could contribute to the smart wearables of the next generation [136].

Conductive yarns were also constructed through a facile continuous electrospinning method by Chen et al. (2022). The composite yarns were based on electrospun PU fibers reinforced by Ag NWs and, except for sufficient conductivity (0.4 kΩ/cm), durability, and mechanical properties, they demonstrated excellent electrothermal features, thus having a potential as an electric heater material for multifunctional wearable devices [137].

Other notable configurations for e-fabrics and e-skins from PU electrospun fibers include thin-film triboelectric nanogenerators (TENG) and sandwich-structured elastic films (ESEFs). In the work of Chen et al. (2017), a soft and stretchable thin-film TENG with two parallel electrodes for versatile energy harvesting was fabricated. The TENG, which combined an electrospun PU nanofiber membrane as a template offering excellent flexibility and Ag NWs as a conductive material, had two working modes: folding and contact separation. In real situations, the TENG could effectively harvest body motion energy when attached on clothes and human skin in both working modes, proving that it can be appropriate as a stretchable power-generation e-skin and wearable energy source [127].

Finally, ESEFs consisting of two electrode layers, namely TPU-AgNW-bridged Ti_3_C_2_T_x_ mats, and one middle dielectric layer (TPU mat), all produced by electrospinning, were proposed in the study of Du et al. (2022). The ESEF-based capacitive e-skins achieved good mechanical characteristics and responded successfully to strain (GF 1.21 kPa^−1^) and pressure (GF = 0.029 kPa^−1^), and can be applied in mechanical stimuli monitoring. This study is really promising, since it suggests a layered thin film for sensory e-skins totally produced through the technique of electrospinning [138].

#### 2.10.2. PVA-Based

PVA was another matrix employed for the fabrication of smart textiles through electrospinning in various works; the most interesting of them are presented followingly in Table 11. First of all, Tebyetekerwa et al. (2018) formed tough, flexible, electroactive yarns for wearable smart textiles by using polyindole (Pind/carbon black (CB)/PVA) nanocomposite nanofibers that were self-assembled and coated onto stainless steel spun textile yarn (SSY) by modified single-nozzle electrospinning. This facile technique tackled the challenges of other coating methods by offering flexibility, good adhesion, and a smart nanoarchitectured design of the nanofiber electroactive materials onto the textile yarn that provided easy ion diffusion sites and led to a satisfactory capacitance [139]. PVA along with PLGA was also used in the study of Peng et al. (2020), who fabricated a biodegradable, antibacterial, and breathable TENG-based e-skin. This three-layered e-skin, the structure of which is depicted in Figure 7c, was formed by sandwiching a silver nanowire (Ag NW) electrode between the top PLGA triboelectric layer and the bottom PVA substrate fabricated by electrospinning, and its capabilities and service life could be tuned by adjusting the concentrations of the materials. The resulting e-skin could assist the human body to navigate, since it achieved the real-time and self-powered monitoring of body movement and paves the way for the optimization of environmentally friendly e-skins [128]. A similar sandwiched TENG structure with the same breathable and antimicrobial properties was also produced by Shi et al. (2021), with the top layer being an electrospun TPU film, the middle an Ag NW electrode, and the bottom a PVA/CS electrospun film (Figure 7d). This nano-structured mat offers a higher surface area, which is an added benefit for sensing uses. Therefore, the e-skin presented a high sensitivity equal to 0.3086 V kPa^−1^ and thermal moisture comfort, and was applied in volleyball as a self-powered wearable sensor for reception statistics and analytics to help athletes [140].

**Table 11:** Bio-based polymers, electrospinning parameters, and processing steps for the fabrication of e-textiles and e-skins destined for various applications.

Material Used for Electrospinning	Optimum Electrospinning Conditions	Process Steps for the Preparation of Smart Textile	Type of Smart Textile	Potential Application	Reference
TPU (matrix), DMF (solvent)	15 kV voltage, 0.8 mL/h feed rate, 30 cm tip-to-collector distance, ∼50 rpm rotating speed of drum	Preparation of PU fibrous non-woven via electrospinning Dip coating of the electrospun PU nonwoven with the conducting polymer poly(3,4-ethylenedioxythiophene):poly(styrenesulfonate) (PEDOT:PSS)	Mechanically stretchable and electrically conductive textile	Wearable electronics	[130]
TPU (matrix), DMF:THF (1:1) (solvent)	20 kV voltage, 3 mL/h feed rate, 15 cm tip-to-collector distance	Fabrication of PU fibrous mat via electrospinning Ultrasonication of reduced graphene oxide (RGO) Fabrication of RGO/PU composite by immersion of PU fibers into RGO solution and ultrasonication	Flexible resistive-type strain sensors	Smart wearable device	[131]
TPU (matrix), DMF:THF (1:3) (solvent)	8 wt%. TPU in DMF:THF, 23 kV voltage, 2.5 mL/h feed rate, 10 cm tip-to-collector distance	Preparation TPU substrate by electrospinning Deposition of carbon nanofibers (CNFs) on TPU by spray coating	Durable, stretchable piezoresistive strain sensor	E-skin for detecting body motions	[132]
TPU (matrix), DMF:THF (1:1) (solvent)	15 wt%. TPU in DMF:THF, 30 kV voltage, 5 mL/h feed rate, 16 cm tip-to-collector distance	Preparation of the TPU fibrous film by electrospinning Fabrication of TPU/carbon nanotubes (CNTs) strain sensor by ultrasonication of CNTs and immersion of TPU film strips into CNTs suspension	High-performance, durable strain sensor for monitoring motion changes	Wearable sensing device, e-skin for human motion monitoring	[133]
PU (matrix), DMF:THF (1:1) (solvent), GO (dopant)	19 kV voltage, 0.5 mL/h feed rate, 15 cm tip-to-collector distance, 400 rpm rotating speed of drum	Fabrication of Ni-coated cotton yarns via electroless deposition Fabrication of GO/PU nanofibers on Ni-coated cotton yarn by electrospinning Wound of GO/PU/Ni-coated cotton yarn around the elastic thread Weaving of a large-area electronic fabric	Highly sensitive and stretchable electronic fabric	Wearable electronic devices	[134]
PU (matrix), DMF:THF (1:1) (solvent), carbon nanotubes—CNT (dopant)	15 kV voltage, 0.3 mL/h feed rate, 15 cm tip-to-collector distance, 400 rpm rotating speed	Fabrication of Ni-coated cotton yarn via electroless deposition Fabrication of core–shell CNT-embedded PU nanofiber sensing yarn via electrospinning	Highly sensitive pressure sensors for human motion and physiological signal monitoring	Smart textiles and wearable electronics	[135]
PU (matrix), DMF:THF (1:1) (solvent), Ag NWs ethanol dispersion (dopant)	∼18 wt% PU in DMF:THF, +20 and −1 kV voltage, 0.3 mL/h feed rate of PU and 3 mL/h feed rate of AgNWs ethanol dispersion	Fabrication of Ag NW-embedded PU nanofiber sensing yarn (AENSY) via electrospinning of PU spinning solution and Ag NW dispersion solution Fabrication of AENSY textile-based sensor and smart textile by encapsulating the AENSY textile-based sensor by two layers of flexible PDMS film and weaving with two AENSYs	Multifunctional sensing yarn textile-based sensor with piezoresistive ability to quantify mechanical deformations	Smart yarns and textiles for personalized healthcare and human–machine interfaces	[136]
PU (matrix), DMF (solvent), AgNWs (additive)	25 wt% PU in DMF, 3 wt% CB in CA, 12 kV voltage, 0.15 mL/h feed rate, 10 cm tip-to-collector distance	Preparation of the Ag NWs/PU composite yarns by electrospinning, spraying, drafting, heating, and winding	High-conductive, flexible, and sensitive yarns	Wearable electronics	[137]
PU(matrix), DMF:THF (2:3) (solvent)	13 wt% PU in DMF/THF solution, 8 kV voltage, 1 mL/h feed rate, 7.5 cm tip-to-collector distance	Electrospinning of PU Doping of PU film with Ag nanowires (NWs)/MWCNTs Formation of micro-patterned PDMS Coating of PDMS with micro-patterned PDMS and Ag NWs/MWCNTs-doped fibers	Stretchable thin-film generator based on electrification for body motion energy harvesting	Wearable energy harvester attached on cloth or self-powered e-skin	[127]
TPU (matrix), THF:DMSO (1:1) (solvent), AgNW and Ti_3_C_2_T_x_ (additives)	12 wt% TPU in THF:DMSO, 2 wt% AgNW and 4 wt% MXene, 15.8 kV voltage, 3 mL/h feed rate, 15 cm tip-to-collector distance	Preparation of TPU/MXene/Ag NW layer via electrospinning Preparation of TPU layer by electrospinning on TPU/MXene/AgNW layer Preparation of TPU/MXene/AgNW layer via electrospinning on TPU layer	Capacitive e-skin for monitoring various mechanical stimuli	Wearable device	[138]
PVA (matrix), water (solvent), Carbon black—CB (conductive material), polyindole–PIND (conductive material)	15 kV voltage, 0.5 mL/h feed rate, 8 cm tip-to-collector distance	Polymerization of Pind (active material) Nanofiber-coated yarns (NCY) fabrication via electrospinning of Pind/CB/PVA solution Preparation of PVA/H_2_SO_4_ gel electrolyte Fabrication of device by assembling NCY sample and coating with PVA/H_2_SO_4_ on PET film	Fully flexible tough electroactive yarns	Wearable smart textiles	[139]
- PVA (matrix), water (solvent) - PLGA (matrix), hexafluoro-2-propanol-HFIP (solvent)	- 8 to 11 wt% PVA in water, 25–28 kV voltage, 15 cm tip-to-collector distance - 6.5 to 9.5 wt% PLGA in HFIP, 9–18 kV voltage, 0.3–1.0 mL/h feed rate, 15 cm tip-to-collector distance	Production of PVA nanofiber scaffold via electrospinning Fabrication of PVA/Ag NW film on PET via vacuum filtration Fabrication of PLGA/PVA/Ag NW film via electrospinning	Breathable, biodegradable, and antibacterial e-skin based on all-nanofiber triboelectric nanogenerators	E-skin with real-time and self-powered monitoring of whole-body physiological signal and joint movement	[128]
- TPU (matrix), DMF: THF (1:1) (solvent); - PVA (matrix of the 3rd layer), water (solvent), CS (matrix of the 3rd layer), acetic acid (solvent)	20 wt% PU in DMF: THF, 10 wt% PVA in water, 2 wt% CS in acetic acid, 26 and 11 kV voltage	Fabrication of PVA/CsurS layer via electrospinning Spray coating of Ag NW solution onto the PVA/CS layer Fabrication of sensing layer via electrospinning of TPU on aluminum foil covered with a PVA/CS/Ag NW film	Flexible, breathable and antibacterial triboelectric nanogenerator (TENG)-based e-skin for self-powered sensing	Wearable electronic device with self-powered sensors	[140]
CA (matrix), DMAc:acetone (2:1) (solvent), CB (dopant)	17 wt% CA in DMAc:acetone, 3 wt% CB in CA, 18 kV voltage, 10 μL/min feed rate, 10 cm tip-to-collector distance	Fabrication of CA/CB nanofibers via electrospinning of CA/CB solution on PU film attached on woven cotton fabric Deacetylysation and gold sputtering	Conductive nanofibres	E-textile, on-skin bio-potential measurement sensor	[141]
gelatin (matrix), PBS (solvent)	10 wt% gelatin in PBS, 15 kV voltage, 2 mL/h feed rate	Preparation of GelMA mat via electrospinning GelMA mat Fabrication of CNTs/graphene/GelMA mat via loading CNTs and graphene on GelMA mat and ultrasonic treatment	Stretchable, conductive, breathable, and moisture-sensitive e-skin	Wound monitoring, home medical diagnosis, and human–machine interactions	[142]
Silk Fibroin and PEO (matrices), water (solvent)	1:89 *w*/*w* PEO/silk fibroin, 18 kV voltage, 20 μL/min feed rate, 20 cm tip-to-collector distance	Fabrication of silk fiber film via electrospinning Preparation of interdigital electrode after fixing a complementary mask via spray coating Fabrication of AgNWs on the silk fiber film forming an intact interdigital electrode	Wearable all-fiber multifunctional sensor	Smart clothing	[129]
PLA and TPU (matrixes), DMF:THF (1:1) (solvent),	25:75 wt% TPU:PLA, 20 kV voltage, 3 mL/h feed rate, 12 cm tip-to-collector distance	5. Fabrication of TPU/PLA electrospun fiber mats 6. Spray coating of the TPU/PLA fiber mats with single-walled carbon nanotubes	Large strain flexible strain sensors with programmable shape memory	Wearable electronics	[143]

#### 2.10.3. Natural-Polymer-Based

In addition, cellulose has also drawn a lot of interest owing to its biocompatibility, biodegradability, and availability, given that it is the most prevalent organic chemical on the planet. Among the various derivatives of cellulose, cellulose acetate was used for the development of conductive fibers for e-textile applications by Thenuwara et al. (2020) thanks to its excellent electrospinnability and chemical, mechanical, and thermal stability. More specifically, in this study, cellulose fibers with CB NPs as conductive media were directly electrospun onto a PU film attached to a cotton woven fabric. The fibers achieved a good electrical conductivity without compromising the properties of the initial fabric, but only those that were in contact with the PU layer were successfully adhered, something that limits their use and is a subject for future research [141].

Furthermore, gelatin is also another natural biodegradable and biocompatible polymer, which is frequently used in would healing. Gelatin can be equipped with extra mechanical stiffness by linking its amino groups with acrylamide groups to form gelatin methacrylate (GelMA). In the work of Li et al. (2022), carbon nanotubes (CNTs) and graphene were loaded in a GelMA mat by electrospinning and an in situ loading method in order to develop a multifunctional, stretchable, conductive e-skin with good water vapour transmission and moisture sensitivity (sensitivity coefficient equal to 12.05) for potential application in wound management and human–machine interactions [142].

Finally, the natural polymer of silk and PEO were utilized as matrixes in electrospinning for the integration of a multifunctional sensor that was successfully used for breathing monitoring in a smart mask, as well as for the recognition of finger bending in a smart glove. While silk fibroin served both as the supporting and the functional sensing component, PEO was employed to prepare a solution suitable for electrospinning, and Ag NWs to offer conductivity during the preparation of the two-layered array, which is analytically described in Table 11 and depicted in Figure 7e. The wearable sensor showed not only a high-pressure sensitivity of 2.27 pF/kPa, but also great flexibility and breathability; some features that make it a good candidate for applications in smart clothing [129].

All the aforementioned advances in smart sensing technology and smart textiles that are based on the operation of electrospinning using bio-based polymers are summed up in Table 11.

## 3. Conclusions

The integration of electrospinning technology with bio-based polymers has led to a new era of innovation, giving rise to protective and smart textiles that showcase technological progress and contribute to environmental conservation. By using electrospinning to produce fibers, these textiles offer fundamental advantages, including a small fiber diameter, easy deformable porous microstructure, ultra-high surface area, and good mechanical flexibility. Additionally, another crucial benefit of electrospun fibers is the capability to adjust their final properties according to their required application by incorporating suitable additional agents into the electrospinning solution that provide or enhance the membrane properties, by tuning the parameters during the process or by functionalizing them through post-spinning treatments, such as coating. These properties respond to the new requirements of the rapid development of Internet of Things (IoT), enabling electrospun fibers to be employed in next-generation smart textiles for applications in health monitoring, robot skin, and intelligent electronic devices. Electrospinning also presents a revolution in protective clothing applications, offering novel barrier characteristics against water, micro-particles, microorganisms, UV irradiation, chemicals, and fire etc., while providing good air permeability and surface adaptability. These protective textiles can be used in sportswear, industrial safety uniforms, military uniforms, facemasks, medical applications, and personal, daily use.

Despite the remarkable advancements, it is crucial to address the environmental impact of electrospun fibers. While various types of fibers can be created through electrospinning, many of these materials are not disposable, posing potential harm to the environment after their service life. Recognizing this, the use of non-toxic biodegradable materials, such as PLA and CA, becomes imperative. These materials, after fulfilling their designated timeframe, completely degrade into non-harmful substances without adverse long-term effects, emphasizing the importance of considering biodegradability in the performance optimization of electrospun smart textiles. Overall, the synergy of electrospinning and bio-based polymers paves the way towards the evolution of protective and smart textiles with enhanced properties.

## Figures and Tables

**Table 1:** Effect of various parameters during electrospinning on the morphology of the produced fibers. (a) Increase up to a critical value, which depends on the polymer system. Increasing the parameter beyond this critical value causes hard ejection of jets from solution. (b) Parameter depending to a great extent on the polymer system. Very high increase leads to the formation of non-uniform, beaded nanofibers [24].

	Electrospinning Parameters	Effects on Fiber Morphology
Solution parameters	Polymer molecular weight ↑	Uniform, beadless electrospun nanofibers Mean fiber diameter ↑
	Polymer concentration ^(a)^ ↑	From beaded to uniform fibers Mean fiber diameter ↑
	Viscosity ^(a)^ ↑	Uniform, beadless electrospun nanofibers Mean fiber diameter ↑
	Surface tension ↑	Greater probability of beaded fibers formation, non-uniform nanofibers Mean fiber diameter ↑
	Conductivity ↑	Uniform nanofibers Mean fiber diameter ↓
Processing parameters	Flow rate ^(b)^ ↑	Mean fiber diameter ↑
	Voltage ^(b)^ ↑	Formation of beads Mean fiber diameter ↓
	Needle-to-collector distance ^(b)^ ↑	Mean fiber diameter ↓
Ambient parameters	Temperature ↑	Mean fiber diameter ↓
	Humidity ^(b)^ ↑	Mean fiber diameter ↑

**Table 2:** Bio-based polymers, materials, and electrospinning parameters for the fabrication of fibers with antimicrobial properties, along with their structure and target application.

Matrix	Solvent	Antimicrobial Agent	Optimum Process Conditions	Structure	Tested Microbes	Target Application	Reference
PU	Tetrahydrofuran (THF):N,N-dimethylformamide (DMF) (1:1, *w*/*w*)	Ag NPs	10 wt% AgNO_3_/DMF, 20 kV voltage, 15 cm tip-to-collector distance	Nanofibers	*E. coli*, *S. typhimurium*	Antimicrobial wound dressing materials	[33]
PU	Tetrahydrofuran (THF):N,N dimethylformamide (DMF) (1:1 *v*/*v*)	GO, Ag NPs	10 wt% PU, 15 kV voltage, 15 cm tip-to-collector	Nanofibers	*E. coli* (79%)	Tissue engineering, wound healing, drug delivery systems	[34]
PU	THF:DMF (1:1 *w*/*w*)	Ag NPs	10% PU with AgNPs, 18 kV voltage, 0.5 mL/h feed rate, 15 cm tip-to-collector	Nanofibers with a diameter of 300 ± 50 nm	*S. aureus* (diameter of inhibition zone 11.4 mm), *E. coli* (diameter of inhibition zone 10.8 mm)	Water filtration, wound dressings	[35]
PU, CS	TFA:DCM (7:3)	CS	PU/CS (85/15), 17 kV voltage, 0.3 mL/h feed rate, 10 cm tip-to-collector	Fibers with a mean diameter of 226 nm	*E. coli* (inhibition zone 4.27 mm)	Air filters, face masks	[36]
PU	DMF (Dimethyl Formamide):THF (Tetrahydrofuran)	Ag NPs	0.015% Ag in 12 wt% PU, 13 kV voltage, 5 mL/h feed rate, 10 cm tip-to-collector distance	Nanofiber mat with a diameter of 150–250 nm	*Klebsiella bacteria* (diameter of inhibition zone 1.1 cm)	Wound dressing	[37]
PU	DMF:THF (1:1.5)	MgO NPs, Ag NPs	PU/MgO/Ag 2 wt, 13 kV voltage, 1 mL/h feed rate, 15 cm tip-to-collector	Nanofibers with a diameter of 116 nm	*E. coli* (inhibition zone 21 mm), *S. aureus* (inhibition zone 30 mm)	Protective clothing, firefighting and industrial protective clothing	[38]
PU	N,N-dimethylformamide (DMF):tetrahydrofuran (THF) (3:1, *v*/*v*)	TiO_2_ NPs	18 kV voltage, 0.5 mL/h feed rate, 15 cm tip-to-collector	Nanofiber mat	*S. aureus* (>99.9%)	Protective clothing, protection layer against chemical and biological warfare agents	[39]
PU	Dimethylformamide:tetrahydrofuran (1:1 *v*/*v*)	Copper (Cu)	20% *w*/*w* PU, 24 kV voltage, 1 mL/h feed rate, 23.5 cm tip-to-collector	Fiber membranes	*E. coli* (98% growth inhibition), *S. aureus* (98% growth inhibition)	Protective clothing applications	[40]
HMW CS	Acetic acid	CS/PEO	HMW CS/PEO (90:10), 30 kV voltage, 0.08 mL/min feed rate, 10 cm tip–target distance	Nanofibrous filter media	*E. coli* (>2 log reduction in 6 h)	Filtration applications, water purification, air filter media	[41]
CS, Hydroxypropyl cellulose (H), PEO (P)	25 wt% acetic acid aqueous solution	Graphene (G)	H: 4.5 wt%/CS: 4.5 wt%/P: 0.75 wt%/G: 0.5 wt%, 25 kV voltage, 0.6 mL/h feed rate, 20 cm tip-to-collector	Nanofibers	*S. aureus*, *E.coli* (survival rates < 0.01%)	Wound dressing	[42]
Trimethylated CS (TMC)-loaded PVA			TMC/PVAl (4:8% *w*/*v*), 16 kV voltage, ~0.5 mL/h, feed rate, 12 cm tip-to-collector	Ultrafine fibers with a diameter of 101 ± 28 nm	*E. coli*, *Pseudomonas aeruginosa*, *Acinetobacter baumannii*, *Candida albicans*	Gloves	[43]
PVA	Water	Zinc oxide (ZnO) NPs	9% by weight PVA/ZnO, 14 kV voltage, 12 cm tip-to-collector	Nanofibers	*S. aurous* (inhibition zone of 10 mm), *E. coli* (inhibition zone of 10 mm)	Medical surgeon	[44]
PVA, CS	DMAc:acetone (60:40, *w*/*w*)	Ag NPs	PVA/CS (92/8 weight ratio) with 1 wt% Ag, 15 kV voltage, 0.1 mL/h feed rate, 10 cm tip-to-collector	Nanofibers	*E. coli* (3 log CFU/mL at 16 h)	Membranes for fuel cell, wound dressing, smart textiles	[17]
CS, PVA	Distilled water with 5% *w*/*w* acetic acid	GO	23 kV voltage, 15 cm tip-to-collector	Nanofibrous membranes with a diameter of 83 ± 10 nm	*E. coli* (inhibition zone of 1.25 mm, CS:PVA:GO = 1:9:0.03), *S. aureus* (inhibition zone of 1.40 mm, CS:PVA:GO = 3:7:0)	Wound dressing	[45]
PVA/Sericin	Water	Clay	10% (*w*/*v*) solution of Sericin/PVA (1:1 wt/wt), Clay concentration 0.75%, 27.5 kV voltage, 0.8 mL/h flow rate, 8 cm tip-to-collector	Nanofibrous mats with an average diameter of 300 nm	*E. coli* (98.3%), *S. aureus* (97%)	Air filtration mask	[46]
PVA	Distilled water	TiO_2_ NPs	11 wt% PVA solution containing 3 wt% TiO_2_ NPs, 20 kV voltage, 0.2 mL/h feed rate, 13 cm tip-to-collector	Nanocomposite fibers with a diameter of 300–400 nm	*S. aureus* (99.3% with 2 wt% TiO_2_), *Klebsiella pneumoniae* (85.3% with 3 wt% TiO_2_)	Sports/outdoor textiles and technical textiles, medical applications	[47]
PVA	Deionized water	Aloe Vera	3% aloe vera, 17 kV voltage, 0.5 mL/h feed rate, 20 cm tip-to-collector	Nanofibers with a diameter of 179.59 ± 0.04312 nm	*S. aureus* (inhibition zone 12.3 mm), *E. coli* (inhibition zone 11.08 mm)	Surgical gowns, gloves, protective clothing	[48]
PVA	Water	Quaternary ammonium salt (IQAS)	7.0 wt% PVA, 0.5% IQAS, 10 kV voltage, 1 mL/h feed rate 12 cm, tip-to-collector, rotation rate 1000 rpm	Nanofibers	*S. aureus* (99.9%), *E. coli* (99.9%)	Wound dressings, textile, food packaging and air filtration, public health settings, surgical equipment	[62]
PCL	DCM:DMF (9:1, *v*/*v*)	Ag, Ascorbyl palmitate (AP)	10% *w*/*v* PCL, PCL/AP (30%), 25 kV voltage, 3.3 mL/h feed rate, 15 cm tip-to-collector, 75 rpm	Nanofibers with a diameter of 380 nm	*S. aureus*	Application in medicine and cosmetics	[49]
PCL	Acetone	Zinc oxide (ZnO) NPs	15 wt% PCL in acetone, PCL/6% ZnO, 18 kV voltage, 1 mL/h feed rate, 15 cm tip-to-collector	Nanocomposite fiber membranes with an average diameter of 2000 nm	*E. coli* (Inhibition zone diameter 9.81 ± 0.8 mm), *S.* *aureus* (Inhibition zone diameter 10.22 ± 1.3 mm)	Tissue engineering scaffold material	[50]
PCL	Chloroform:methanol (4:1 *v*/*v*)	Hydrophobic carbon quantum dots (hCQDs)	15% *w*/*v* PCL with 0.3% *w*/*w* hCQDs, 25 kV voltage, 1.0 mL/h feed rate, 13 cm tip-to-collector distance	Fibers with no beads	*S. aureus* (8.2 × 10^1^ cfu/cm^2^), *Listeria monocytogenes* (1.9 × 10^1^ cfu/cm^2^), *E. coli* (6.3 × 10^1^ cfu/cm^2^), *Klebsiella pneumoniae* (9.4 × 10^3^ cfu/cm^2^)	Wound dressing	[51]
PCL	Chloroform (CHF):N,N-Dimethylformamide (DMF ) (4:1)	MgO NPs	15% (*w*/*v*) of PCL, 25 kV voltage, 1 mL/h feed rate, 26 cm tip-to-collector	Nanofiber membrane	*S. aureus* (inhibition zone 25.3 ± 0.6 mm), *E. coli* (13.5 ± 0.7 mm)	Facemasks	[52]
PCL	Acetic acid:formic acid (2:1)	ZnO NPs	9 wt% PCL, 55 kV voltage, 10 cm tip-to-collector	Nanofibers with a thickness of 100 μm	*S. aureus*, *E. coli*, *Candida parapsilosis*, *Neurospora crassa*	Biomedical applications, including in a protective filter layer	[63]
PCL/CS oligosaccharides (COS)	Formic acid:acetic acid (7:3 *v*/*v*)	Quercetin (Qe)	15% *w*/*w* (COS/PCL), 32 kV voltage, 0.77 mL/h feed rate, 15 cm tip-to-collector	Nanofibers with a diameter of 119.1 ± 24.6 nm	*E. coli*, *S. aureus*	Wound healing, wound dressings for burn injuries	[53]
CA	DMF:acetone (1:2, wt/wt)	ZnO/AgNPs	10 wt% ZnO/AgNPs, 18 kV voltage, 0.06 mm/min feed rate, 15 cm tip-to-collector	Nanofibers	*E. coli* (inhibition zone of 1.27 ± 0.17 mm), *S. Aureus* (inhibition zone of 1.32 ± 0.15 mm)	Antibacterial wound dressings	[54]
CA	Acetone:dimethylformamide (DMF) (2:1 *v*/*v*)	Lemon myrtle essential oil (LMEO)	17% *w*/*v* CA with 5–20 wt% LMEO, 17.5 kV voltage, ~0.5 mL/h feed rate, 15 cm tip-to-collector	Nanofibers with a diameter of 493–515 nm	*E. coli and S. aureus* (100% elimination)	Active packaging or wound-dressing materials	[55]
CA/PEO	DMF:DCM (1/1 *w*/*w*)	Rutin	8 wt% CA, 1.6 wt% PEO, 2 wt% Rutin, 15 kV voltage, 1 mL/h feed rate, 15 cm tip-to-collector	Fiber membrane	*S. aureus* (97.2%), *E. coli* (98.5%)	Applications at pharmaceutical and cosmetic industries	[56]
PBS	PBS was dissolved in chloroform with 15% *w*/*v* ratio	Ag NPs	PBS solution to PVP-capped AgNPs solution was 4:1 *v*/*v*, 1.16 wt% Ag, 20 kV voltage, 50 μL/min feed rate, 15 cm tip-to-collector	Fiber mats with a diameter of 291.9 ± 82.4 nm	*S.aureus* (99%), *E. coli* (99%)	Biocidal membranes	[57]
PHB	2,2,2-trifluoroethanol (TFE)	ZnO NPs	1 wt% ZnO, 25 kV voltage, 2.5 mL/h feed rate, 25 cm tip-to-collector	Uniform fibers with a fiber diameter of 380 μm	*S. aureus* (2.10 CFU/mL), *E. coli* (3.20 CFU/mL)		[58]
PLA	Chloroform:N, N Dimethylformamide:tetrahydrofuran (1:1:1)		Drug blended 8 wt%/v PLA, 15 kV voltage, 1 mL/h feed rate	Fiber with a diameter of 375 nm	*B. cereus* (inhibition zones 7.84 ± 0.28 mm), *L. monocytogenes* (inhibition zones 23.16 ± 1.89 mm), *E. coli* (inhibition zones 28.33 ± 1.44 mm), *S. typhi* (inhibition zones 22.66 ± 0.76 mm)	Antimicrobial activities	[59]
Silk fibroin/gelatin	98% formic acid	GO–Ag NPs	SF/GT 15% *w*/*w*, 18 kV voltage, 15 cm tip-to-collector	Nanofiber film	*E. coli*	Biomedical applications	[60]
PHBV, PBAT	Chloroform:DMF (7:3)	TiO_2_ NPs	1.0 wt% TiO_2_, 17 kV voltage, 1.5 mL/h feed rate, 15 cm tip-to-collector	Nanofiber membrane	*S. aureus* (98.5%), *E. coli* (99.7%)	Biomedical application, medical protection	[61]

**Table 3:** Bio-based polymers, materials, and electrospinning parameters for the fabrication of fibers with filtration properties along with their structure and target application.

Matrix	Solvent	Filtration Agent	Optimum Process Conditions	Structure	Filtration Efficiency	Target Application	Reference
HMW CS	Acetic acid	CS/PEO	HMW CS/PEO (90:10), 30 kV voltage, 0.08 mL/min feed rate, 10 cm tip–target distance	Nanofibrous filter media with a diameter of 65 nm	Aerosol filtration efficiency 70%	Filtration applications, water purification, air filter media	[41]
PVC/PU	THF:DMF (1:9, *w*/*w*)		PVC:PU (8/2, *w*/*w*), 28 kV voltage, 2.5 mL/h feed rate, 20 cm tip-to-collector distance	Fiber with diameter of 960 mm	Air filtration efficiency 99.5% towards NaCl APs (diameter of 300–500 nm)	Filtration technology, vehicle engine air filter, cabin air filter, and gas turbine dust collection systems	[66]
CA	Acetone:N,N-dimethylacetamide (DMAc) (3:1 *w*/*w*)		75–80 kV voltage, 0.2 mL/h feed rate, 20 cm tip-to-collector, 11.2 rpm	Fiber with a diameter of 23.8 μm	100% air filtration efficiency towards NaCl (d_p_ 120 nm) and diethyl hexyl sebacate (DEHS) APs	Air filters	[15]
CA, Cetylpyridinium bromide (CPB)	Acetic acid/Distilled water (3:1 *v*/*v*)		21% (*w*/*v*) of CA and 0.5% (*w*/*v*) of the surfactant CPB, 18 kV voltage, 0.7 mL/h feed rate, 10 cm tip-to-collector	Nanofibers with average diameter of 239 nm	100% filtration efficiency for APs which can include black carbon (BC)	Facial masks, indoor air filter materials in air conditioning equipment	[67]
PLA	DCM:DMAC (10:1, *w*/*w*)		5 wt% PLA, 15 kV voltage, 5.5 m/min feed rate, 12 cm tip-to-collector	Fiber with a diameter of 273.6 ± 41.0 nm	Air filtration efficiency 99.997% towards NaCl APs (average diameter of 260 nm)	Respiratory protection, indoor air purification, and other filtration applications	[69]
PLA	DMF:Acetone at a ratio of 4:6 *v*/*v*		25 kV voltage, 1 mL/h feed rate, 20 cm tip-to-collector	Nanofibers with mean diameter of 646 ± 368 nm	Filtration efficiency 80.5% (single layer) Filtration efficiency 95.0% (dual layer)	Face mask filter	[70]
Piezoelectric PLA	DCM:DMF (7:3 *v*/*v*)		10 wt% PLA, 15 kV voltage, 1 mL/h feed rate, 15 cm tip-to-collector, 120 rpm	Fibers with an average diameter of 500 nm	Air filtration efficiency 99% for PM 2.5 and 99.8% for PM 10	Air cleaning systems and personal air purifier applications	[71]
PLA	DMF	Activated carbon (A.C.)	10% PLA-8% A.C, 30 kV voltage, 3.0 mL/h feed rate, 15 cm tip-to-collector	Nanofibers with an average diameter of 80–240 nm	Bacterial Filtration Efficiency (BFE) ≥ 98%, Differential Pressure (Delta P) < 5.0 mm H_2_O/cm^2^, Submicron Particle Filtration Efficiency ≥ 98%, Penetration Resistance (Synthetic Blood) 160 mm Hg	Protective equipment production and filtration applications	[67,72]
Sericin/PVA	Water	Clay	Clay concentration 0.75%, 10% (*w*/*v*) solution of Sericin/PVA (1:1 wt/wt), 27.5 kV voltage, 0.8 mL/h feed rate, 8 cm tip-to-collector	Nanofibrous mats with an average diameter of 300 nm	Filtered 0.725 mg/m^3^/s of PM of 1 μm Filtered 1.407 mg/m^3^/s of PM of 2.5 μm. Filtered 4.175 mg/m^3^/s of PM of 10 μm	Air filtration mask	[46]
PCL	Chloroform (CHF)/N,N-Dimethylformamide (DMF) (4:1)	MgO NPs	15% (*w*/*v*) of PCL, 25 kV voltage, 1 mL/h feed rate, 26 cm tip-to-collector	Nanofiber membrane with a thickness of 0.147 mm	Filtration efficiency 99.4%	Facemasks	[52]
PU/CS	TFA:DCM (7:3)	CS	PU/CS (85/15), 17 kV voltage, 0.3 mL/h feed rate, 10 cm tip-to-collector	Fibers with a mean diameter of 226 nm	75–100% for KCl particles with a size of 10–1000 nm	Air filters, face masks	[36]
PU	N,N-dimethylformamide (DMF)		10 wt% PU in N,N-dimethylformamide, 25 kV voltage, 0.5 mL/h feed rate, 15 cm tip-to-collector, 680 rpm	Fibers with an average diameter of 200 nm	Air filtration efficiency 100% for particle size > 0.6 μm Air filtration efficiency 92–95% for particle size < 0.6 μm	Protection against hazardous chemicals, and for specialized medical and military uses	[73]
Gelatin powder	Acetic acid:DI water (80:20 *v*/*v*)		Area density of 3.43 g/m^2^ (thickness = 16 μm), 18–20 kV voltage, 0.6 mL/h feed rate, 10 cm tip-to-collector	Fibers with a diameter of 69 ± 17 nm	Removal efficiency of 99.51 ± 0.23% for PM_2.5_. Removal efficiency of 99.63 ± 0.11% for PM_10–2.5_	Green air-filtering materials	[74]
Recycled PET	Trifluoroacetic acid (TFA):Dichloromethane (DCM) (70:30)		10 wt% PET, 5 μL/min feed rate, 25 cm tip-to-collector	Fiber with a diameter of 0.4 μm	Smoke filtration. 43.7 times its own weight of absorbed smoke components	Industrial filters	[75]

**Table 4:** Bio-based polymers, materials, and electrospinning parameters for the fabrication of fibers with air permeability properties along with their structure and target application.

Matrix	Solvent	Additional Agent	Optimum Process Conditions	Structure	Air Permeability	Target Application	Reference
PU	N,N-dimethylacetamide (DMAc)		12% *w*/*w* PU, 13 kV voltage, 10 cm tip-to-collector distance	Fibers with an average diameter of 1.45 μm—PU web/fabric	5.50 × 10^−1^ cm^3^/s/cm^2^	Protective and specialty textiles	[76]
PCL based PU	N,N-dimethylformamide:tetrahydrofuran (1:1 *v*/*v*)		6.5 kV voltage, 1.0 mL/h feed rate	Electrospun web	7.00 cm^3^/s/cm^2^	Intelligent clothing material	[81]
PU	THF:N,N-dimethylformamide (60:40 *v*/*v*)		13 wt%/v of commercial PU, 13 kV voltage, 13 cm tip-to-collector distance, 150 rpm rotating speed, 400 mm/min traverse speed of the drum, 12 h of electrospinning	Fibers with an average diameter of 480 nm	3.56 × 10^−7^ cm^3^/s/cm^2^	Protective clothing	[82]
PU	THF:N,N dimethylformamide (DMF) (60:40, *v*/*v*)		14 kV voltage, 0.6 mL/h feed rate, 17 cm tip-to-collector distance, 4 h electrospinning of PU	Fiber with a diameter of 447 mm	4.20 cm^3^/s/cm^2^	Sportswear, protective clothing, and orthopedic dressing	[79]
PU	DMF:THF (3:2 *v*/*v*)		12% (*w*/*w*) PU, 12 kV voltage, 0.2 mL/h feed rate, 13 cm tip-to-collector distance	Uniform nanofiber web with an average diameter of 890 nm	2.5 cm^3^/s	Army combat, sports uniforms	[77]
PU	N,N-dimethylformamide (DMF)	Hydrophobic silica gel (HSG)	18 wt% PU/HSG, 3 wt% HSG with respect to the polymer PU, 15 kV voltage, 0.6 mL/h feed rate, 20 cm tip-to-collector distance, 300 rpm rotating rate of the collector	Fibrous membranes with an average diameter of 331 nm	9.20 × 10^−1^ cm^3^/s/cm^2^	Protective clothing, water purification, tissue engineering	[80]
FPU	DMF:THF (weight ratio of 1:2)	SiO_2_ NPs	FPU 18 wt%, SiO_2_ 1 wt%, 18 kV voltage, 0.5 mL/h feed rate, 15 cm tip-to-collector distance	Superamphiphobic nanofibrous membranes with an average diameter of 915 nm	1.48 × 10^−1^ cm^3^/s/cm^2^	Protective clothing	[83]
PU, FPU	DMF		4 wt% PU, 0.5 wt% FPU, 20 kV voltage, 2 mL/h feed rate, 15 cm tip-to-collector distance	Microfibrous membranes with a thickness of 30 μm	8.46 × 10^−1^ cm^3^/s/cm^2^	Protective clothing	[88]
PVC/PU	THF:DMF (1:9, *w*/*w*)		PVC:PU (8/2 *w*/*w*), 28 kV voltage, 2.5 mL/h feed rate, 20 cm tip-to-collector distance	Fiber with a diameter of 960 mm	15.41 cm^3^/s/cm^2^	Applications in filtration technology	[66]
PU, PAMPS	THF:DMF (Nozzle 1: 60:40, Nozzle 2: 0:100)	GO	Nozzle 1: 6 *w*/*w*% PU, 12 kV voltage, 0.5 mL/h feed rate, 14 cm tip-to-collector distance Nozzle 2: 20 *w*/*w*% PAMPS, 14 kV voltage, 0.1 mL/h feed rate, 14 cm tip-to-collector distance 100 rpm speed of collector, 16 cm/min traverse motion of collector	Nanofibrous membrane	1.57 cm^3^/s/cm^2^	Protective clothing, wound dressing	[84]
PU	Dimethylformamide (DMF) (>98%):tetrahydrofuran (THF) (4:1 *v*/*v*)	SiO_2_ NPs	8.2 wt% PU, 5 wt% SiO_2_, 14 kV voltage, 0.2 mL/h feed rate, 18 cm tip-to-collector distance	Webs with diameters of 600 to 700 nm	211.60 cm^3^/s/cm^2^	Textile laminate materials	[85]
PU	Water	Polycarbodiimide (PCD) and long-chain alkyl polymer (LAP) emulsions (PCE and LAE), PEO	9 wt% PCE, 15 wt% LAE, 40 kV voltage, 4 mL/h feed rate, 22 cm tip-to-collector	Nanofibrous membranes with an average fiber diameter of 548 nm and thickness of 150 ± 5 μm	1.99 cm^3^/s/cm^2^	Medical hygiene, wearable electronics, water desalination, and oil/water separation.	[8]
PCL	Chloroform (CHF):N,N-Dimethylformamide (DMF) (4:1 *v*/*v*)	Functionalization with MgO NPs	15% (*w*/*v*) of PCL in CHF/DMF, 25 kV voltage, 1 mL/h feed rate, 26 cm tip-to-collector	Nanofiber membrane with a diameter of 2.02 μm	2.00 cm^3^/s/cm^2^	Facemasks	[52]
PLA	N,N-dimethylformamide (DMF):dichloromethane (DCM) 8:2 m/m	AlCl_3_	1 wt% AlCl_3_, 25 kV voltage, 1 mL/h feed rate, 17 cm tip-to-collector	Fibers with a diameter of 559 nm	10.90 cm^3^/s/cm^2^	Filtration, separation, biomedical, personal protection	[86]
PVA	Water		10 wt% PVA solution, 15 kV voltage, 0.5 mL/h feed rate, 10 cm tip-to-collector	Nanofibers with a diameter of 12–13 μm	28.55 cm^3^/s/cm^2^	Face masks	[87]

**Table 7:** Bio-based polymers, materials, and electrospinning parameters for the fabrication of fibers with UV protection properties along with their structure and target application.

Matrix	Solvent	Additional Agent	Optimum Process Conditions	Structure	UV Protection	Target Application	Reference
PVA	Water	TiO_2_ NPs	11 wt% PVA solution with 2 wt% TiO_2_ NPs, 20 kV voltage, 0.2 mL/h feed rate, 13 cm tip-to-collector	Layered fabric system of nanocomposite fibers with a diameter of 300–400 nm and web area density of 3.0 g m^−2^	UPF (ultraviolet protection factor) = 50+, UV-A transmittance (%) = 8.8, UV-B transmittance (%) = 0.3	Sports/outdoor textiles and technical textiles, medical applications	[47]
PVA	Water	ZnO NPs	10 wt% PVA, 9 wt% ZnO, 14 kV voltage, 12 cm tip-to-collector	Nanofibers with a diameter of ~0.5 nm	UV transmission (%) = 0	Medical surgical gowns	[44]
Lignin/PVA	Water	Lignin	50 wt% lignin, 25 kV voltage, 0.4 mL/h feed rate, 10 cm tip-to-collector	Nanocomposite fiber webs with fiber diameter of 95–230 nm and web area density of 3.0 g m^−2^	UPF = 50+, UV-A transmittance (%) = 0.1, UV-B transmittance (%) = 0.1	Functional fibers and textiles	[110]
PU, PAN	N,N-dimethylacetamide (DMAc)	Core material: TiO_2_ NPs. Coating modification with fluorinated acrylic copolymer (FAC), 2-hydroxy-4-n-octoxybenzophenone (UV531)	10 wt% PAN/PU (8/2 mass ratio), 1 wt% TiO_2_ NPs, 2 wt% FAC, 0.5 wt% UV531, 30 kV voltage, 1 mL/h feed rate, 20 cm tip-to-collector distance	Nanofibrous membranes with an average diameter of ~350 nm	UPF = 1485	High-altitude garments, protective clothing, covering materials, self-cleaning materials, and other medical products	[102]
PU	Dimethylformamide:tetrahydrofuran (1:1 *v*/*v*)	Modification with zinc and copper (Cu) salt	20% *w*/*w* PU, 24 kV voltage, 1 mL/h feed rate, 23.5 cm tip-to-collector, modification with: 0.036 mol Cu salt in 100 mL water and 0.0183 mol NH_4_Cl	fiber membranes with an average fiber diameter of 1.58 ± 0.22 μm	UPF= 50+, UV-A Blocking (%) = 99.99, UV-B Blocking (%) = 100	Protective clothing	[40]
Cotton cellulose	LiCl:DMAc (8.0% *w*/*v* LiCl)	After electrospinning: immersion in Ce(NO_3_)_3_ 6H_2_O/Hexamethylenetetramine (HMT)	1.15% activated cellulose (*w*/*v*), 21 kV voltage, 0.8 mL/h feed rate, 12 cm tip-to-collector distance	Nanofibers with a diameter of 100–200 nm	UV absorbance = 2.5–3.2 at 200–350 nm	Medical, military, biological, and optoelectronic industrial fields	[107]
PLA	Dichloromethane (DCM):N,N-Dimethylformamide (DMF) (7:3 *w*/*w*)	Collection of PLA fibers on PLA fabric with TiO_2_ NPs	10% PLA (*w*/*v*), 18 kV voltage, 0.03–0.08 mL/min feed rate, 15 cm tip-to-collector	Fibers with an average diameter of ~0.5 μm	UV-B transmittance (%) = 14–16	Multifunctional personal protective materials	[104]

**Table 8:** Bio-based polymers, materials, and electrospinning parameters for the fabrication of fibers with chemical protection properties along with their structure and target application.

Matrix	Solvent	Additional Agent	Optimum Process Conditions	Structure	Thermal Properties	Target Application	Reference
**Temperature resistance/thermal regulation**							
PEG, PCL	DCM:DMF (70:30, *v*/*v*) for shell layer precursor solution DCM:DMF (50:50, *v*/*v*) for core layer precursor solution		10 wt% PCL, 70 wt% PEG, 18 kV voltage, 2.20 mL/h feed rate of the shell solution, 0.22 mL/h feed rate of the core solution 13 cm tip-to-collector	Fibers with average diameter of 1200 nm	Melting enthalpy: 39.5 × 10^3^ J/kg Thermal conductivity: 0.1662 W/mK	Smart fabrics, medical items, and biosensors	[116]
PEG, PVA	Distilled water		PEG:PVA (7:3), 18–20 kV voltage, 1 mL/h feed rate	Fiber with an average diameter of 780 ± 31 nm	Heat enthalpy: 78.10 × 10^3^ J/kg	Smart textile and energy storage systems	[113]
PVA, dodecanol (DD)	Ultrapure water		10 wt% PVA, 8 wt% DD, 15 kV voltage, 0.5 mL/h feed rate, 16 cm tip-to-collector	Fibers	Phase transition enthalpy: 67 × 10^3^ J/kg Good thermal cycle stability with a loss rate of melting enthalpy as low as 0.3%		[117]
Siliceous PU (SIPU), PU	DMAC:Acetone (2:3 *v*/*v*)	Stearic acid (SA)	SIPU solution 11 wt%, SIPU:PU (1:1), SA 30 wt%, 25.5 kV voltage, 22 cm tip-to-collector	Fiber with an average diameter of 230 nm	Good thermal stability up to the temperature of 200 °C Medium latent heat enthalpy of phase change of 40 × 10^3^ J/kg	Outdoor protective clothing, medical clothing, intelligent clothing, and military products	[98]
Melamine, PVA	Formaldehyde		8 wt% PVA, 15–21 kV voltage, 0.5 mL/h feed rate	Fibers with a diameter of 0.53 ± 0.13 μm	Heat resistance up to 370 °C	High-temperature-resistant filter	[118]
PCL	Chloroform (CHF):N,N-Dimethylformamide (DMF) (4:1)	MgO NPs	15% (*w*/*v*) PCL, 25 kV voltage, 1 mL/h feed rate, 26 cm tip-to-collector	Nanofiber membrane	Facial temperature Nose: 34.1 °C Mouth: 33.5 °C Cheek: 30.6 °C	Facemasks	[52]
PEG, PCL	HFIP	Gelatin, Circumin	20 kV voltage, 25 μL/min feed rate, 11 cm tip-to-collector, 300 rpm	Fiber with a diameter of 220–370 nm	Latent heat of 61.7 × 10^3^ J/kg and reliable energy absorption release cyclability over 100 heating–cooling cycles.	Medical textiles for biomedical dressing applications	[114]
PEG, PLA	Dichloromethane (DCM):N,N-dimethyl-formamide (DMF) (4:1, *v*/*v*)		PEG:PLA 200:100 mass ratio, 13 kV voltage, 3 mL/h feed rate, 15 cm tip-to-collector	Fibers with an average diameter of 1540 ± 204 nm	Melting enthalpy: 74.70 × 10^3^ J/kg, degradation temperature higher than 320 °C	Applications in smart textiles, medicine cares, and electric devices for thermal energy storage and temperature regulation	[115]
PU/PEG	PU was dissolved in THF:DMF (1:1 *w*/*w*) PEG was dissolved in DMF		25 wt% PU, 70 wt% PEG, 11–14 kV voltage, 2 mL/h feed rate of the shell solution, 0.5 mL/h feed rate of the core solution, 13 cm tip-to-collector	Fibers with an average diameter of 3.23 ± 0.58 μm	Melting enthalpy: 60.40 × 10^3^ J/kg	Next-generation of thermal regulation textiles	[112]
**Flame retardancy**							
PU	DMF:THF (1:1.5)	MgO NPs	PU/MgO 4 wt%, 13 kV voltage, 1 mL/h feed rate, 15 cm tip-to-collector	Nanofibers with a diameter of 116 nm	Flame Retardancy: After flame time: 26.51 s After glow time: 7.67 s Char length: 8.5 cm UL 94 rating: V1	Protective clothing, firefighting, and industrial protective clothing	[38]
PLA, DiDOPO (derivative of 6H-dibenz(C,E) (1,2)oxaphosphorin-6-oxide (DOPO))	DCM:DMF (7:3 *v*/*v*)		12% *w*/*v* PLA, 30% *w*/*w* DiDOPO, 20 kV voltage, 2 mL/h feed rate, 15 cm tip-to-collector	Fiber with a diameter of 2–12 μm	UL 94 rating: V0 Limited oxygen index (LOI): 27.3%		[68]

**Table 9:** Bio-based polymers, materials, and electrospinning parameters for the fabrication of fibers with chemical properties along with their structure and target application.

Matrix	Solvent	Additional Agent	Optimum Process Conditions	Structure	Chemical Properties	Target Application	Reference
PVA	Distilled water	TiO_2_ NPs	11 wt% PVA solution containing 3 wt% TiO_2_ NPs, 20 kV voltage, 0.2 mL/h feed rate, 13 cm tip-to-collector	Layered fabric system of nanocomposite fibers with a diameter of 300–400 nm and web area density of 3.0 g m^−2^	Formaldehyde decomposition efficiency = 80% (15 h UV irradiation) Ammonia deodorization efficiency = 32.2% (2 h UV irradiation)	Sports/outdoor textiles and technical textiles, medical applications.	[47]
CS	Trifluroacetic acid:Dichloromethane (2:8 ratios)	ZnO NPs	13% CS, 65 kV voltage, 13.5 cm distance between spinning electrodes, 8 rpm rotation of drum spinning electrode	Nanoweb with an average fiber diameter of 700–715 nm	DCP (Dichloropropane) penetration time = 270 s (=> chemical protection of 40 h)	Next-generation lightweight NBC suit for soldiers	[103]
CA/PEO	DMF and DCM (1/1 *w*/*w*)	Rutin	8 wt% CA, 1.6 wt% PEO, 2 wt% Rutin in membrane (0.2 wt% in solution), 15 kV voltage, 1 mL/h feed rate, 15 cm tip-to-collector	Fiber membrane with average fiber diameter of ~700 nm	Antioxidant activity = 98.3% (*p* < 0.05)	Applications in pharmaceutical and cosmetic industries	[56]

**Table 10:** Bio-based polymers, materials, and electrospinning parameters for the fabrication of fibers with shape memory properties along with their structure and target application.

Matrix	Solvent	Additional Agent	Optimum Process Conditions	Structure	Shape Memory Properties	Target Application	Reference
PCL-based PU	DMF		3.0–12.0 wt% PU:DMF, 12–25 kV voltage, 0.04–0.1 mm/min feed rate, 15 cm tip-to-collector distance	Nanofibers with average diameter of 50–700 nm	R_rec_: 98%, R_f_: 80%	Smart materials	[123]
PCL-based PU	N,N-dimethylformamide:tetrahydrofuran (1:1)		4 wt% PU, 6.5 kV voltage, 1.0 mL/h feed rate, 5–20 cm tip-to-collector distance	Films with a thickness of 40 μm	R_ret_: 86%, R_rec_: 85%	Protective and thermally intelligent textiles	[81]
PU (shell), PEG (core)	THF:DMF (1:1 *w*/*w*) for PU DMF for PEG		30 wt% PEG, 11–14 kV voltage, 2 mL/h feed rate of the shell solution, 0.5 mL/h feed rate of the core solution, 13 cm tip-to-collector	Fibers	Full shape recovery at 23 s at 60 °C	Next generation of thermal regulation textiles	[112]
Poly(x-pentadecalactone) hard segments (PPDL) and PCL/CA	Chloroform		5–15 wt% PDLCL:Chloroform, 15 kV voltage, 20 μL/min feed rate, 15 cm tip-to-collector distance	Fibers with an average diameter of 1.8–3.1 μm	R_rec_: 89–95%, R_f_: 82–83%	High-performance filter media, protective clothes, composites, drug delivery systems, and biomaterial scaffolds for tissue engineering	[124]
PCL, epoxy composite	CH_2_Cl_2_:DMF (4:1)		15 wt% PCL, 15 kV voltage, 0.002 mm/s feed rate of PCL, 0.0005 mm/s feed rate of epoxy composite, 18 cm tip-to-collector distance	Nanofibers	R_rec_ only in 6.2 s (T = 70 °C)	Smart structures, biomedical, or other practical fields	[125]
Bisphenol A diglycidyl ether (DGEBA), PCL	Acetone:DMF (3:1 *v*/*v*)	Iodonium salt	PCL:DGEBA 50:50 wt ratio, 9 kV voltage, 0.30 mL/h feed rate, 15 cm tip-to-collector distance	Fibers with an average diameter of 1.04 μm	R_rec_: 88–100%, R_f_: 95–99%	Smart separation membranes	[126]
Bisphenol A diglycidyl ether (DGEBA), PCL	Acetone:DMF (3:1 *v*/*v*)	Iodonium salt	8 kV voltage, 0.20 mL/h feed rate, 20 cm tip-to-collector distance	Fibers with an average diameter of 1.05 μm	R_rec_: >95%, R_f_: >95%	Tissue engineering, drug delivery, composites, or separation membranes	[122]
PLA, Lactic acid oligomer (OLA)	CHCl_3_:DMF (4:1 *v*/*v*)		80 wt% PLA, 20 wt% OLA, 20 kV voltage, 0.50 mL/h feed rate, 14 cm tip-to-collector distance	Fibers with an average diameter of 620 ± 121 nm	R_rec_: 100% (T = 45 °C) R_rec_: >79% (T = 40 °C) R_f_: >95% (T = 45 °C) R_f_: =98% (T = 40 °C)	Biomedical application	[120]

## Abbreviations

Definition	Abbreviation
Aerosol particles	APs
Carbon black	CB
Carbon nanotubes	CNTs
Carbon nanowires	CNWs
Cellulose acetate	CA
Chitosan	CS
Fluorinated polyurethane	FPU
Graphene oxide	GO
Nanoparticles	NPs
Phase-changing materials	PCMs
Poly (2-acrylamido-2-methylpropane sulfonic acid)	PAMPS
Poly (3-hydroxybutyrate)	PHB
Poly (3-hydroxybutyrate-co-3-hydroxyvalerate)	PHBV
Polyacrylonitrile	PAN
Poly (butylene-adipate-co-terephthalate)	PBAT
Poly (butylenes succinate)	PBS
Polycaprolactone	PCL
Polyethylene glycol	PEG
Polyethylene Oxide	PEO
Polyethylene terephthalate	PET
Polylactic acid	PLA
Polymethyl methacrylate	PMMA
Polyurethane	PU
Polyvinyl alcohol	PVA
Polyvinyl chloride	PVC
Polyvinylidene fluoride	PVDF
Thermoplastic Polyurethane	TPU
Waterborne Polyurethane	WPU
